# Cardanol and Eugenol Based Flame Retardant Epoxy Monomers for Thermostable Networks

**DOI:** 10.3390/molecules24091818

**Published:** 2019-05-10

**Authors:** Yvan Ecochard, Mélanie Decostanzi, Claire Negrell, Rodolphe Sonnier, Sylvain Caillol

**Affiliations:** 1 ICGM, UMR 5253–CNRS, Université de Montpellier, ENSCM, 240 Avenue Emile Jeanbrau, 34296 Montpellier, France; yvan.ecochard@enscm.fr (Y.E.); melanie.decostanzi@gmail.com (M.D.); sylvain.caillol@enscm.fr (S.C.); 2 C2MA, IMT—Mines Alès, 6, avenue de Clavières, 30100 Alès, France; rodolphe.sonnier@mines-ales.fr

**Keywords:** cardanol, eugenol, epoxy, thermoset, flame retardant

## Abstract

Epoxy materials have attracted attention for many applications that require fireproof performance; however, the utilization of hazardous reagents brings about potential damage to human health. Eugenol and cardanol are renewable, harmless resources (according to ECHA) that allow the achievement of synthesis of novel phosphorylated epoxy monomers to be used as reactive flame retardants. These epoxy building blocks are characterized by ^1^H NMR and ^31^P NMR (nuclear magnetic resonance) and reacted with a benzylic diamine to give bio-based flame-retardant thermosets. Compared to DGEBA (Bisphenol A Diglycidyl Ether)-based material, these biobased thermosets differ by their cross-linking ratio, the nature of the phosphorylated function and the presence of an aliphatic chain. Eugenol has led to thermosets with higher glass transition temperatures due to a higher aromatic density. The flame-retardant properties were tested by thermogravimetric analyses (TGA), a pyrolysis combustion flow calorimeter (PCFC) and a cone calorimeter. These analyses demonstrated the efficiency of phosphorus by reducing significantly the peak heat release rate (pHRR), the total heat release (THR) and the effective heat of combustion (EHC). Moreover, the cone calorimeter test exhibited an intumescent phenomenon with the residues of phosphorylated eugenol thermosets. Lastly, the higher flame inhibition potential was highlighted for the phosphonate thermoset.

## 1. Introduction

Epoxy thermosets are among the most used polymers in the world due to their excellent chemical, thermal, mechanical and dielectric properties. They are found in many applications such as coatings, adhesives, composites and high performance materials [1,2,3]. Despite this broad range of applications, most of the epoxy monomers used are obtained from oil-based resources and may cause several issues in terms of health and environmental concerns. Indeed, Bisphenol A diglycidyl ether (DGEBA), which results from the reaction between bisphenol A and epichlorohydrin, is the major epoxy precursor [4]. However, bisphenol A is an endocrine disruptor and a “carcinogenic, mutagenic and reprotoxic” (CMR) substance [5,6]. Furthermore, epichlorohydrin is also a classified CMR substance. Thus, in terms of sustainable development, synthesizing biobased and less harmful aromatic monomers for epoxy thermosets has become a major challenge in the last few years [7,8,9,10,11], especially to replace DGEBA. Hence, the interest around renewable phenols has recently grown in both industry and academia. Indeed, several works have been reported in the literature on vanillin [12], lignin [13,14], cardanol [7,8], eugenol [15,16] and their derivatives. With different functionalized sites, biobased phenols can be transformed into new monomers to create very promising thermosets with high renewable content and high thermo-mechanical properties. Indeed, their phenol functions can be used to obtain dimers or trimers, increasing the functionality of monomers. Furthermore, biobased phenols bearing unsaturation can be easily oxidized into epoxy monomers without the use of epichlorohydrin. In this context, eugenol and cardanol seem to be very attractive molecules for the synthesis of epoxy monomers. Eugenol is generally obtained either from cloves (of which the main product is clove oil: 80 wt.%) or from lignin by enzymatic reactions [17]. It has been used for high rigidity epoxy thermosets for different applications requiring a high heat resistance and low flammability [16]. Cardanol is also interesting because it’s a non-edible phenolic oil obtained from cashew nutshell, a side-product of the cashew industry, and is often used to obtain soft materials due to its aliphatic chain. It is considered to be one of the most promising renewable aromatic by-products, relatively cost-effective and available.

In order to increase both aromatic density and thermal stability, biobased epoxy trimers have been obtained using a phosphorus linker (POCl_3_) and phenols by our team [18]. Furthermore, phosphorus atoms are well known to improve flame retardancy in condensed phases and/or gas phases [19,20,21]. Many studies were led on phosphorus-containing epoxy materials and their ability to improve flame retardant properties [22,23,24]. Cardanol was already used as an additive for biobased flame retardants by Pillai et al. through phosphorylation and oligomerization [25]. The obtained prepolymer presents high reactivity with amines, aldehydes and isocyanates and promising properties as a flame-retardant additive. Fu et al. showed also the efficiency of cardanol–based zirconium phosphate as a flame-retardant additive for epoxy composite [26]. However, a reactive optimization way was developed by Wazarkar and Sabnis using phosphorylated cardanol based dimers as curing agents [27]. Thermogravimetric analyses, UL94 and limiting oxygen index (LOI) tests on coatings highlight, in this study, the good flame-retardant properties and a synergistic effect of phosphorus and sulphur atoms mainly on the char production. Around 2 wt.% of phosphorus and 4.6 wt.% of sulphur lead to UL94 V0 rating, but LOI is only slightly enhanced from 20 to 22. Alternately, the flame retardant properties of tri(epoxized-eugenyl)phosphate (TEEP) cured with diaminodiphenylmethane (DDM) have been analyzed by Miao et al. and compared to DGEBA based thermosets [28]. TEEP-DDM thermoset reaches UL94 V0 rating, a LOI of 31.4 and a low pHRR of 119 W/g in PCFC. Only a few studies have been carried out to show the fireproof and mechanical effectiveness of phosphorus functions associated with biophenols to propose new thermosets with a high added value.

The aim of this work is to design biobased epoxy monomers based on eugenol and cardanol to prepare thermosets with a high phosphorus content and two oxidation degrees of phosphorylated functions. Indeed, the presence of the phosphorus atom inside the network allows to obtain excellent intumescent properties without the loss of thermo-mechanical properties, the major drawback of the addition of flame-retardant additives to thermosets. Furthermore, the reactive way prevents migration of flame-retardant compounds out of the network. This original grafting of cardanol and eugenol monomers owing to phosphorylated functions increases the functionality of biophenol monomers. Trimerization or dimerization is followed by the epoxidation of double bonds in mild conditions, before curing with a benzylic diamine (*meta*-xylylendiamine: MXDA) leading to materials with a high biobased content. We finally report the characterizations of those biobased high performance thermosets to evaluate the structure-properties relationship. Consequently, the impact of the phosphonate or the phosphate, the functionality as well as the influence of the structure on flame-retardant and thermo-mechanical properties have been studied.

## 2. Results and Discussion

The synthesis of four monomers with various structures, in terms of their epoxy functionality, aromatic density and phosphorus functions, was performed in order to determine the influence of these different parameters on both the thermo-mechanical and flame-retardant properties of the final materials. For this purpose, three phosphate, and one phosphonate monomers were synthesized. Phosphorus containing flame retardants exhibit different kinds of modes of action, such as flame inhibition in the gas phase, and char enhancement and intumescent in the condensed phase [29,30]. More specifically, phosphonate and phosphate groups are well known for their flame retardant activity mainly in the condensed phase as char promoters, while phosphinate and phosphine oxides have a significant gas phase activity but a lower ability to promote char [31,32]. Furthermore, phosphates are reported to have the best efficiency in char promotion [32].

### 2.1. Monomers Characterization

**Scheme 1:**
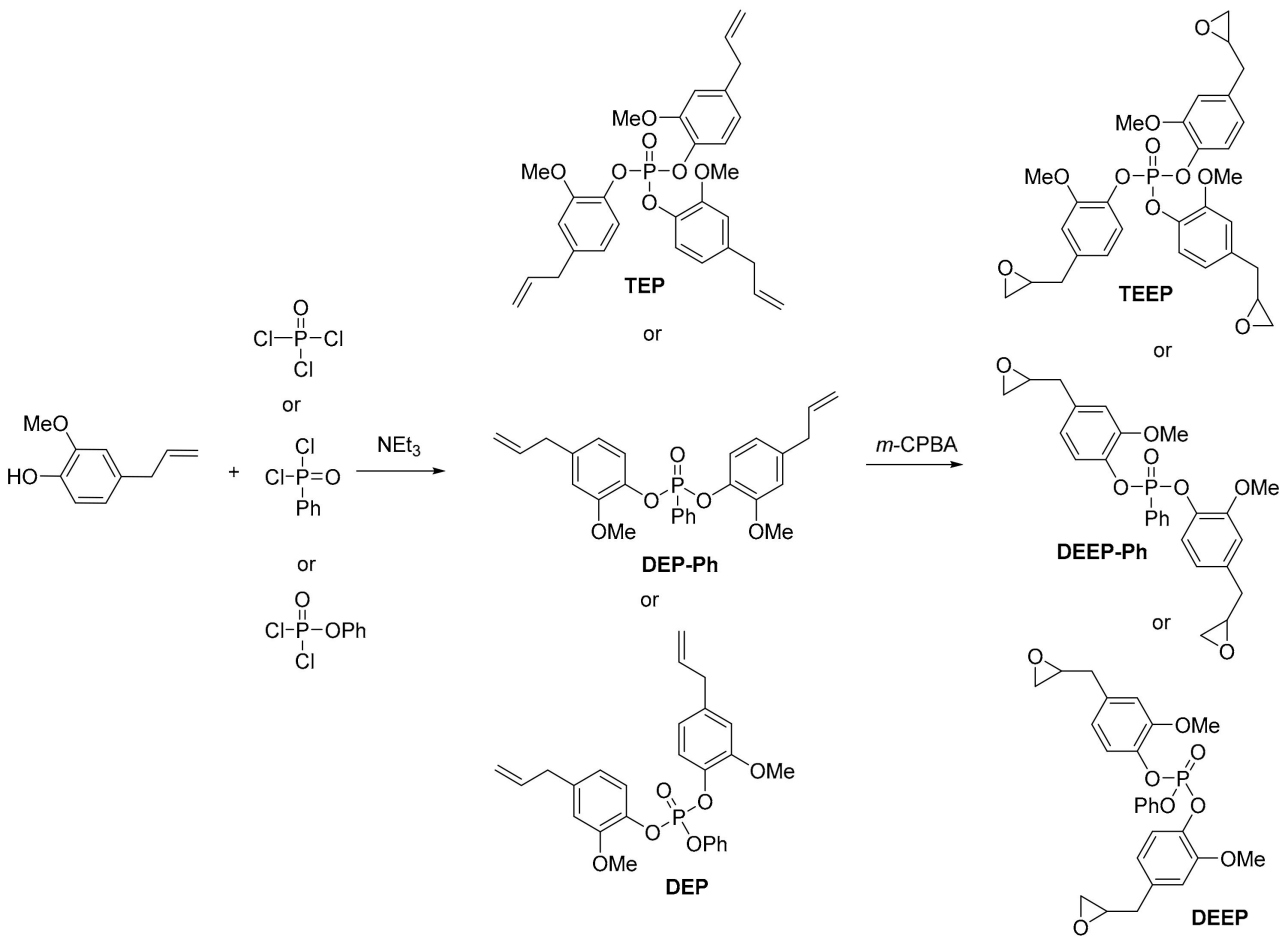
Synthesis pathway for eugenol-based monomers.

**Scheme 2:**
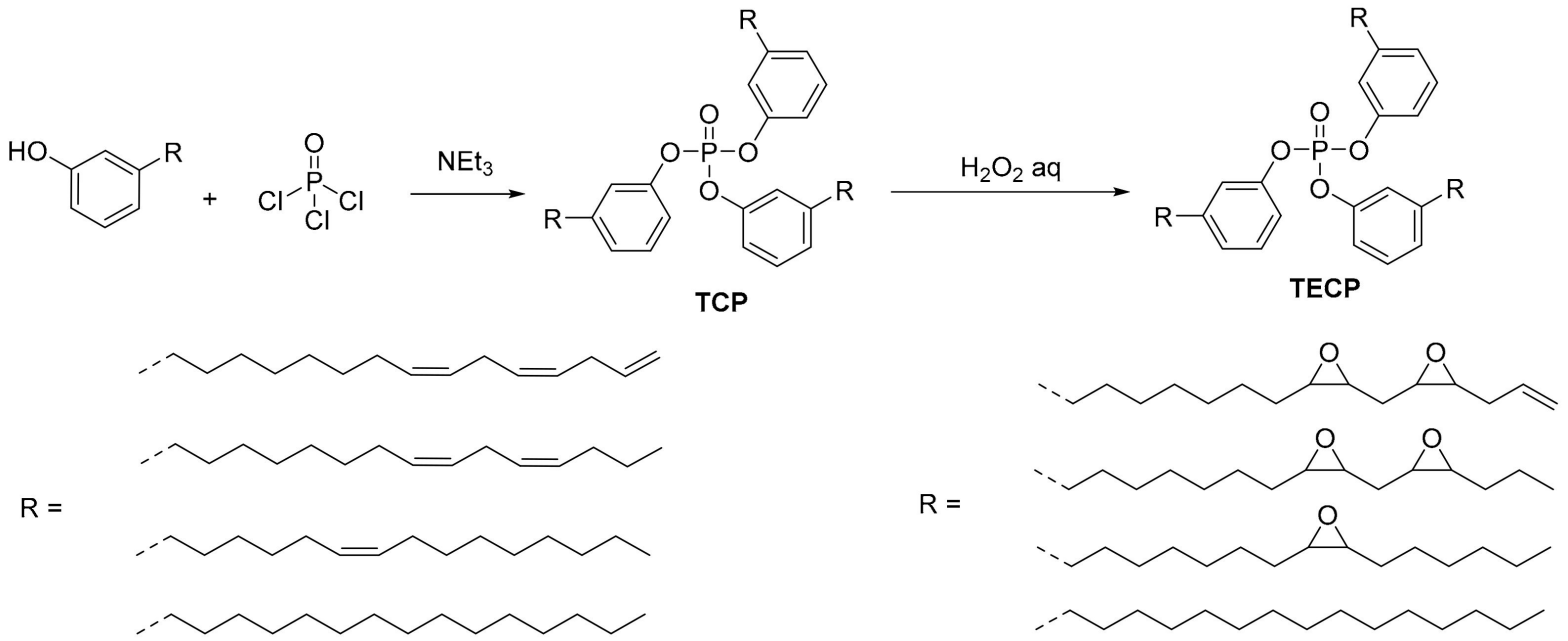
Synthesis pathway for cardanol-based monomer. (Cardanol is a mixture of different molecules containing from 0 to 3 unsaturations).

Three monomers from eugenol (Scheme 1) and one from cardanol (Scheme 2) have been synthesized following our previous synthesis pathway [18]. In both cases, the first reaction is a nucleophilic substitution of the chloride by the phenol group. With the use of POCl_3_ the monomer formed has a functionality of 3 while the use of POCl_2_Ph or POCl_2_(OPh) leads to a functionality of 2. The epoxidation reaction was then performed. *Meta*-chloroperoxybenzoic acid (*m*-CPBA) was used for the epoxidation reaction of allylic function of eugenol, whereas hydrogen peroxide was used for the epoxidation of internal unsaturations of cardanol. Due of the toxicity of *m*-CPBA, the use of hydrogen peroxide was favored when relevant. In fact, hydrogen peroxide is used for the epoxidation of internal unsaturations whereas *m*-CPBA is used for terminal unsaturations [33].

**Figure 1:**
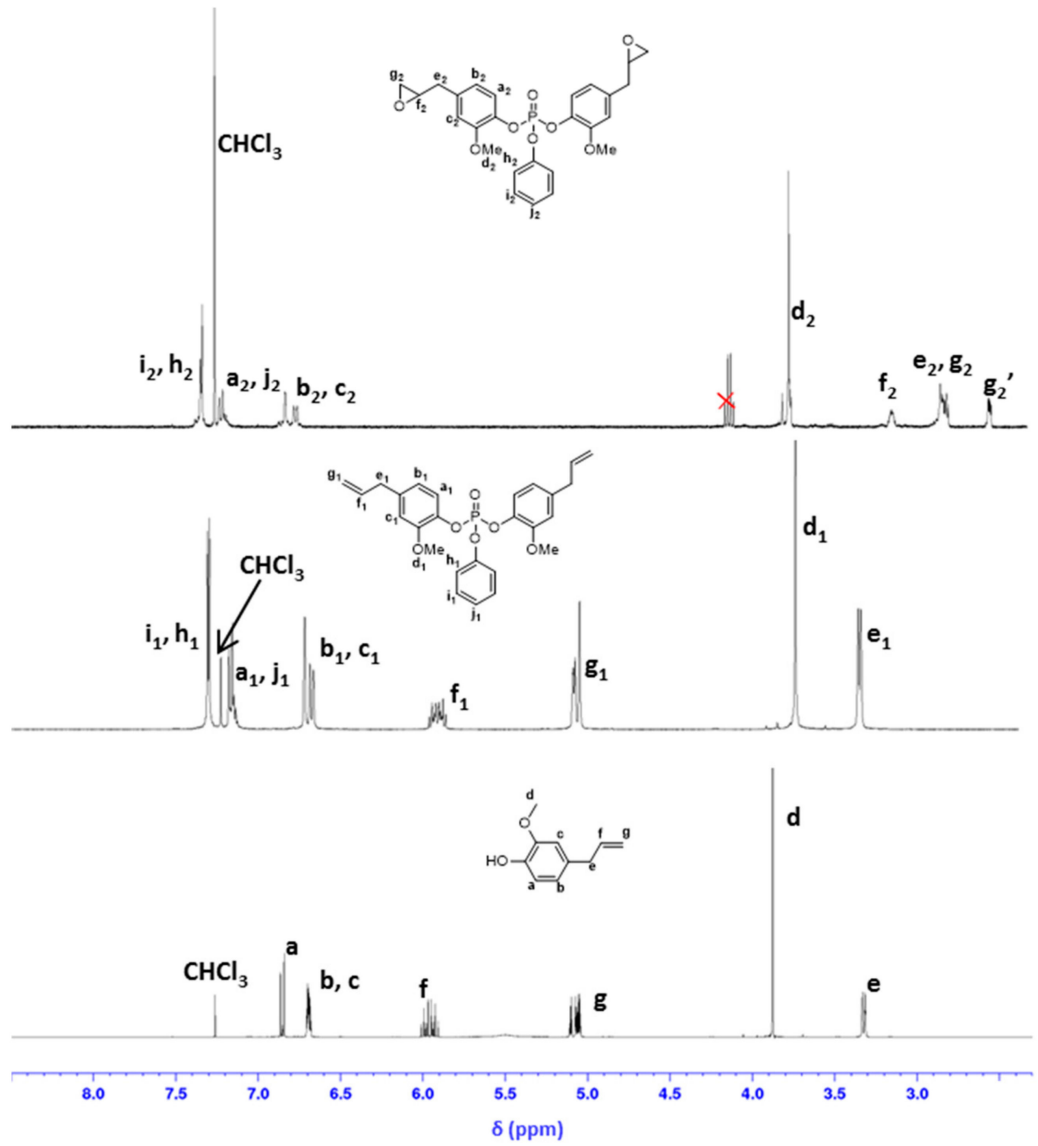
Stacked ^1^H NMR spectra of: Eugenol (bottom), DEP (middle) and DEEP (top) in CDCl_3_.

The structures of the three new monomers, DEEP, DEEP-Ph and TECP (TEEP was already reported [18]) were characterized by ^1^H and ^31^P NMR. The ^1^H NMR of the TEEP monomer and all the ^31^P NMR are in the Supplementary Materials (Figures S1–S10). Figure 1 shows the stacked ^1^H NMR spectra of eugenol, dieugenylphosphate (DEP) and di(epoxized-eugenyl)phosphate (DEEP). In the spectra of eugenol, the signals at 6.7 and 6.9 ppm were assigned to aromatic protons *a, b* and *c*. The protons of the double bond can be found at 5.0 (*g*) and 5.9 ppm (*f*) as multiplets. The methoxy protons (*d*) were localized at 3.8 ppm and finally the allylic protons (*e*) were observed at 3.3 ppm. In the spectrum of DEP, only a few changes are observed. Indeed, the aromatic protons (*a*) are deshielded when the hydroxyl function is grafted onto the phosphate. Furthermore, the signals of the benzene protons were observed at 7.1–7.2 ppm (*j*_1_) and at 7.3–7.4 ppm (*i*_1_ and *h*_1_). Finally, in the spectrum of DEEP, the disappearance of both olefinic and allylic protons was observed. They were replaced by the epoxy protons (*f*_2_ and *g*_2_) and by the glycidyl protons (*e*_2_) respectively at 3.0, 2.9 and 2.6 ppm. All of these observations confirmed the chemical structure of DEEP epoxy monomer in a quantitative yield.

**Figure 2:**
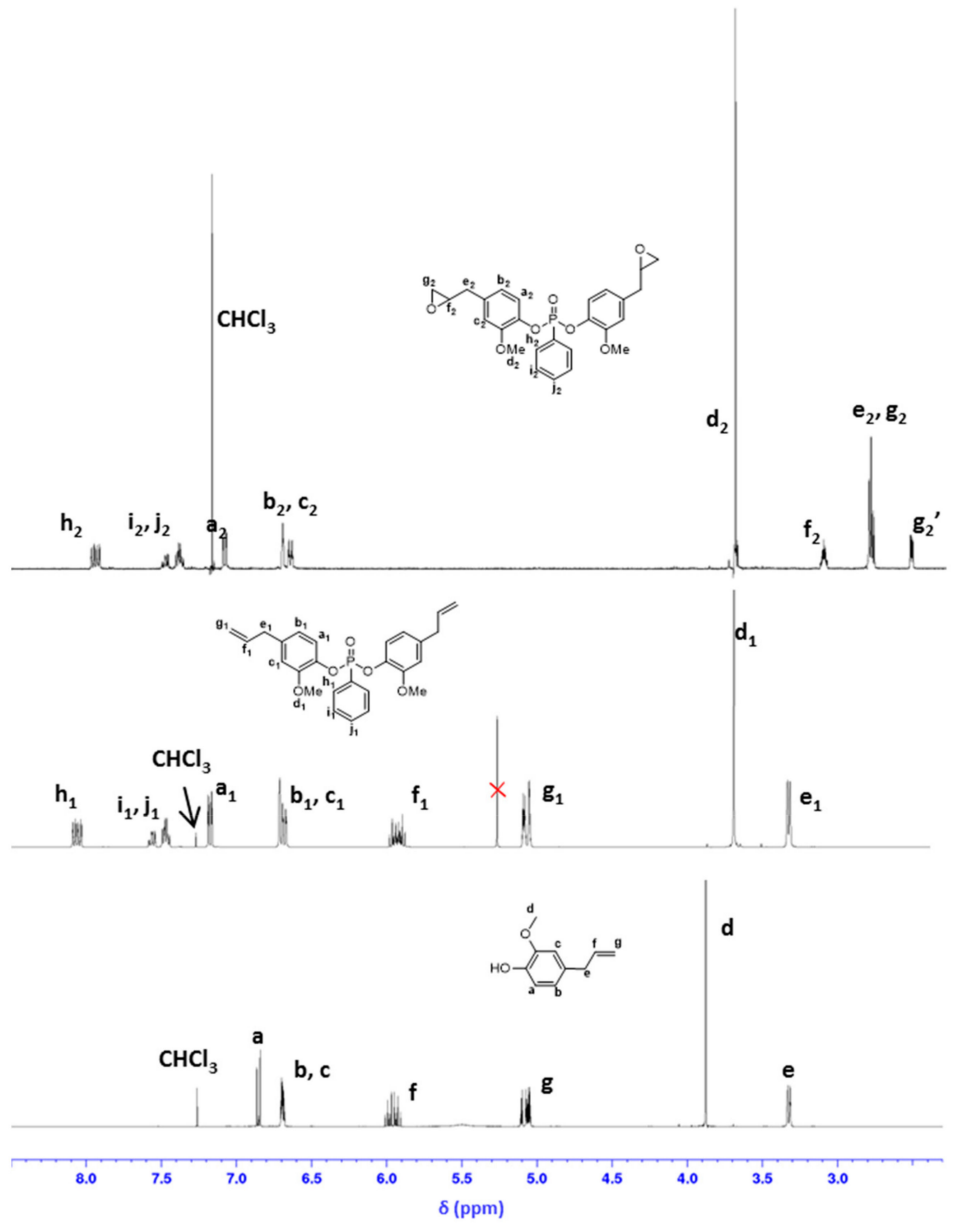
Stacked ^1^H NMR spectra of: Eugenol (bottom), DEP-Ph (middle) and DEEP-Ph (top) in CDCl_3_.

Figure 2 shows the stacked ^1^H NMR spectra of eugenol, dieugenylphosphonate (DEP-Ph) and di(epoxized-eugenyl)phosphonate (DEEP-Ph). In the spectrum of DEP-Ph, the aromatic protons (*a*) are deshielded when the hydroxyl function is grafted onto the phosphonate. Furthermore, the signals of the benzene protons were observed at 7.4–7.7 ppm (*i*_1_ and *j*_1_) and at 8.0–8.1 ppm (*h*_1_). Finally, in the spectrum of DEEP-Ph, the disappearance of both olefinic and allylic protons was observed. They were replaced by the epoxy protons (*f*_2_ and *g*_2_) and by the glycidyl protons (*e*_2_) respectively at 3.0, 2.9 and 2.6 ppm.

**Figure 3:**
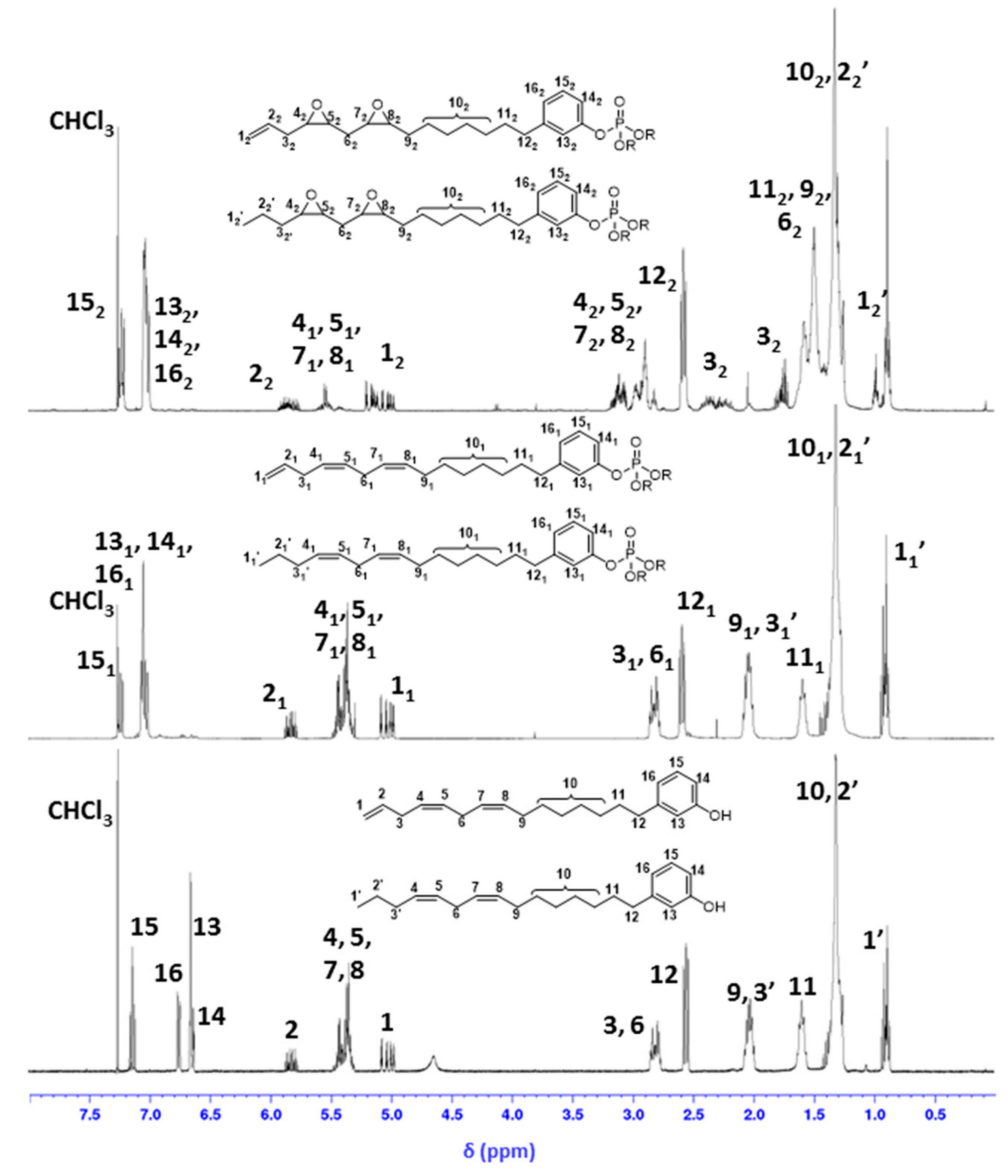
Stacked ^1^H NMR spectra of: Cardanol (bottom), TCP (middle) and TECP (top) in CDCl_3_.

Figure 3 shows the stacked ^1^H NMR spectra of cardanol, TCP and TECP. In the spectrum of cardanol, the signals at 7.1, 6.8 and 6.7 ppm were assigned to aromatic protons *13*, *14*, *15* and *16*. The protons of the terminal unsaturation can be found at 5.8 (*2*) and 5.0 ppm (*1*) whereas the internal unsaturations were localized at 5.4 ppm (*4*, *5*, *7* and *8*). The protons in α position of two double bonds were observed at 2.7 ppm (*6* and *3*) whereas the protons in α position of only one double bond can be found at 2 ppm (*9* and *3′*). The benzylic protons are localized at 2.5 ppm (*12*). The protons at 1.6 and 1.3 ppm were assigned to the protons of the aliphatic chain (*2′*, *10* and *11*). Finally, the saturated terminal protons (*1′*) were observed at 0.9 ppm. In the spectrum of TCP, the main change comes from the deshielding of the aromatic protons (*13_1_*, *14_1_*, *15_1_* and *16_1_*) when the hydroxyl function is grafted onto the phosphate. Finally, the internal unsaturations were epoxidized using hydrogen peroxide. Indeed, this reagent is well known to hardly epoxidize the terminal unsaturations. This result was confirmed by ^1^H NMR. Indeed, 29% of terminal and 88% of internal double bonds were respectively epoxidized. Furthermore, the signals of the formed epoxy functions were observed at 3.0 ppm. The COSY ^1^H-^1^H NMR was used to assign all the protons (see Supplementary Materials—Figure S11).

It is demonstrated that ^31^P NMR confirmed the presence of phosphate and phosphonate functions with peaks at −16.5, −15.9, −17.6 and 13.0 ppm for DEEP, TEEP, TECP and DEEP-Ph respectively.

### 2.2. Characterization and Properties of Thermosets

These four epoxy monomers have been reacted with *meta*-xylylenediamine (MXDA) in order to synthesize epoxy thermosets. Both phosphonate and phosphate linkers allow to bind two or three molecules together, and to form epoxy monomers with high aromatic density and functionality. The reaction of these monomers with short diamine leads to rigid thermosets.

MXDA has been selected for its compatibility with the original phosphorus-containing epoxy monomers and the stiffness conferred by the aromatic ring. Furthermore, a new biobased synthesis pathway of MXDA has been recently reported by Jérôme et al. [34]. In order to compare materials with industrial epoxy thermosets, DGEBA have been chosen for its large utilization at industrial scale and for structure similar to the synthetized monomers.

A stoichiometric ratio of two epoxy functions for one amine function was used for the synthesis of all materials. The EEW was calculated via ^1^H NMR titration with an internal standard. The results are respectively 195 g/eq for TEEP, 310 g/eq for DEEP, 241 g/eq for DEEP-Ph and 240 g/eq for TECP.

The degree of cross-linking of the networks was characterized with gel content measurement (Table 1). The calculation methods were described in the experimental section. Gel content corresponds to the residual mass of materials after immersion in THF and drying. With values up to 99%, no free species remain in the network and the cross-linking is complete for eugenol-based materials. The lower GC values of TECP materials are explained by the presence of non-crosslinked oligomer chains. Indeed, internal epoxy functions of the cardanol chain are less reactive than terminal epoxy functions of eugenol. More probably, cardanol is a mixture of several molecules and some of them contain saturated, non-reactive aliphatic chains that probably remain unreacted in the network.

DSC measurements have been performed on each material. The curves are summarized in Supplementary Materials (Figure S12). High glass transition temperature values are obtained with eugenol monomers. In fact, short monomers with aromatic rings led to highly cross-linked and stiff thermosets. On the opposite, the dangling chain of cardanol confers flexibility to the materials and allowed to obtain materials with lower T_g_ values. We observed some differences for T_g_ values which are 84, 98 and 109 °C of TEEP, DEEP and DEEP-Ph materials, respectively. Values of T_g_ are linked to the mobility of the network and are thus influenced by its rigidity but also linked to the mobility of each segment. With kneecap movements, methoxy groups have shown an important influence on the glass transition temperature of materials [35,36]. This can explain the drop of T_g_ for TEEP material which possesses three methoxy groups against two for the DEEP material. This also explains the high T_g_ of the DGEBA material around 115–120 °C [37,38]. Indeed, the weak mobility of the DGEBA monomer increases the stiffness of the network. Due to the phosphorus-carbon bond in the phosphonate group, the DEEP-Ph material presents less mobility than the DEEP material with its phosphorus-oxygen-carbon bonds. Thus, the DEEP-Ph material presents the highest T_g_ of the eugenol-based materials.

**Figure 4:**
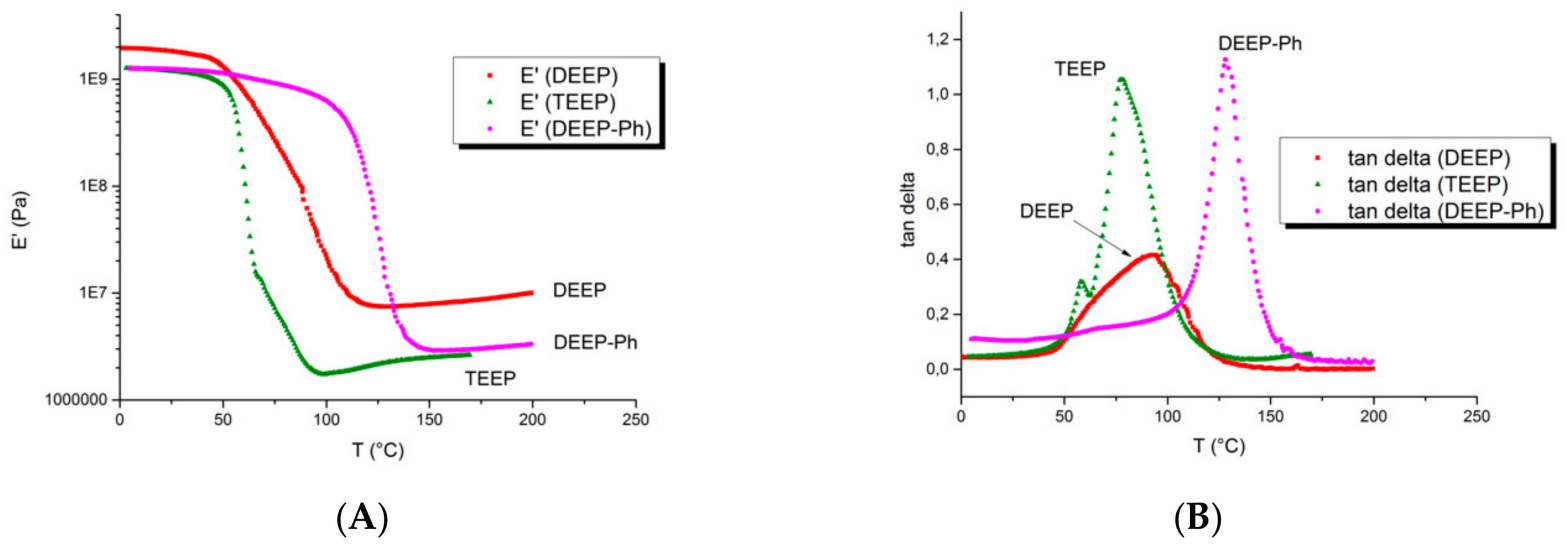
E′ (**A**-left) and tan δ (**B**-right) of DMA curves for eugenol-based materials (red: DEEP; green: TEEP; magenta: DEEP-Ph).

**Figure 5:**
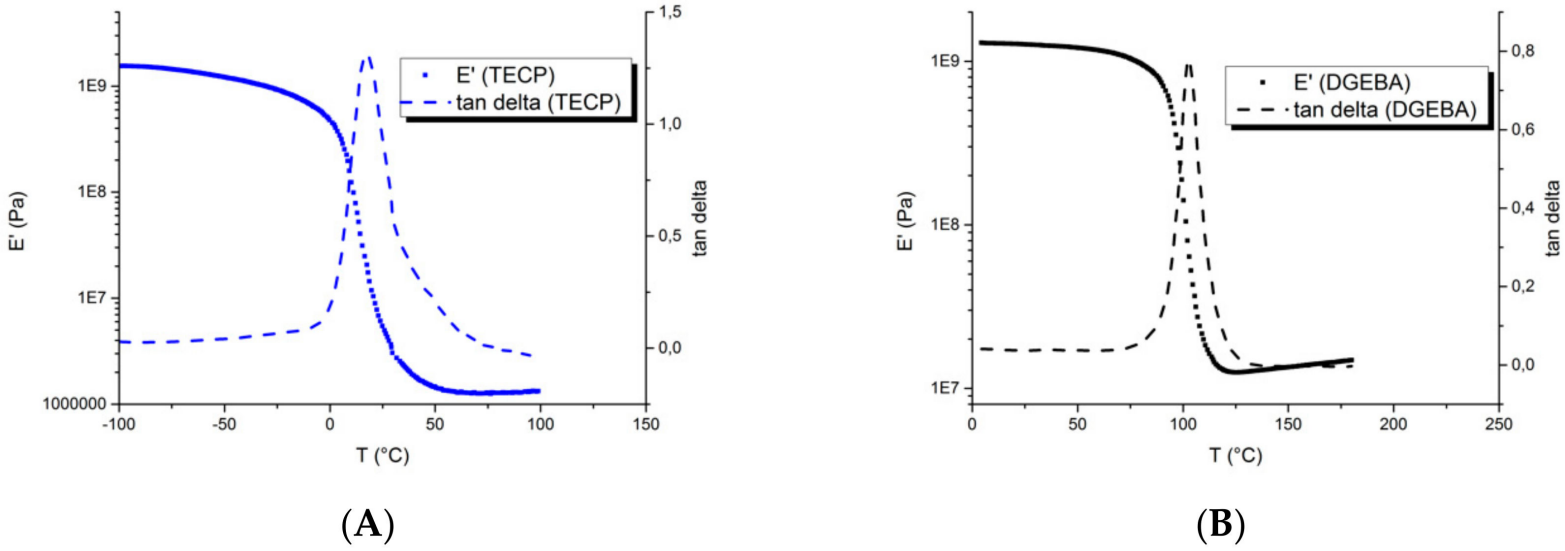
E′ (**A**-left) and tan δ (**B**-right) of DMA curves for cardanol-based and DGEBA-based materials (blue: TECP; black: DGEBA).

The thermo-mechanical properties of the different materials have been compared by DMA and the results are outlined in Table 1. The storage modulus (E′) and tan δ as function of the temperature are shown in Figure 4 and Figure 5. The alpha-transition temperatures have been measured at the maximum of the tan δ for each material. With slightly higher values, T_α_ values followed the same trend than T_g_ values measured by DSC. Narrow peaks of tan δ for all materials, except the one with DEEP, show a good homogeneity. According to the theory of rubber elasticity [39,40], the molecular weight between the crosslinking-nodes of a cured epoxy network is proportional to its storage modulus in the rubbery region. Thus, the cross-linking density (ν′) has been calculated on the rubbery domain at 150 °C for the eugenol-based materials and 100 °C for TECP. The storage modulus (E′) on the rubbery domain is dependent on the stiffness of materials and follows the same trend as the cross-linking density (ν′). As explained for DSC measurements, DGEBA leads to the stiffest materials and presents the highest values of E′ _rubbery_, ν′ and T_α_. According to the values of E′ _rubbery_ and ν′ the stiffness of eugenol-based materials ranged as follows: DEEP > DEEP-Ph > TEEP. The order of T_α_ for DEEP and DEEP-Ph is inverted compared to E′ _rubbery_ measurements but the tendency of those results is similar to T_g_ for which the DSC measurement and explanation has been given earlier. Such a difference in cross-linking density could therefore be explained by the presence of side reactions. Transesterification can occur with phosphate groups and high temperature of curing can promote this side reaction [41]. Phosphate groups being less stable than phosphonate groups are more able to create new bonds via this side reaction that could explain a higher cross-linking density with DEEP. Side reactions may also cause heterogeneity in materials, which could explain the broad tan δ peak with DEEP. Indeed, higher values of E′ _rubbery_ could be expected for TEEP but the higher flexibility brought by methoxy groups as well as the space between chains impact the network, and this can explain these lower values. Finally, TECP was the softest material due to its long and dangling aliphatic chains with a E′_rubbery_ of 1.3 MPa at T_α+50_ °C and a crosslinking density of 140 mol/m^3^.

### 2.3. Flame Retardancy

**Figure 6:**
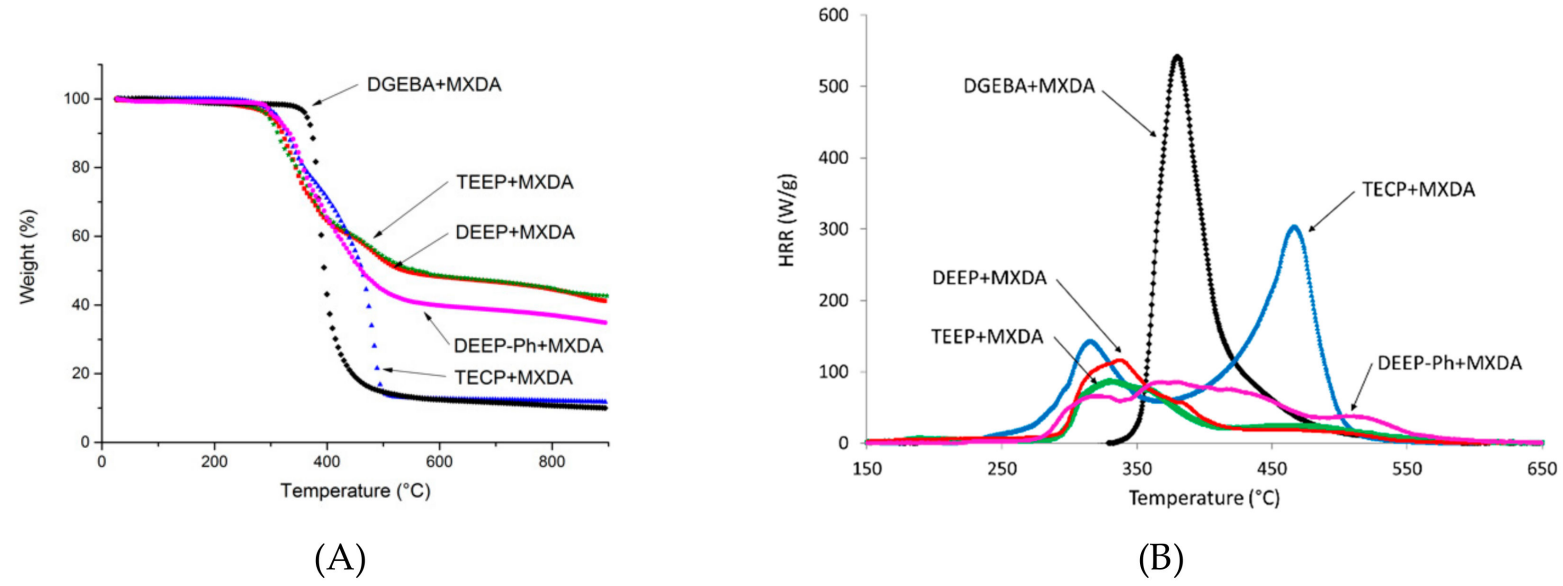
(**A**-left) Thermograms of the cured materials (**B**-right) Heat release rate versus temperature in pyrolysis-combustion flow calorimeter tests.

Firstly, TGA measurements were carried out under nitrogen atmosphere to characterize thermal stability of the materials and the curves are shown in Figure 6A. All these degradations occurred over 300 °C given by the values of T_d5%_. These degradation temperatures were similar but slightly lower to those obtained from DGEBA. Furthermore, the char yields of DEEP, DEEP-Ph and TEEP-based materials are higher than char yields of TECP-material because they present higher aromatic densities than the cardanol formulation. In all cases, the char yields are higher compared to DGEBA-based thermoset.

The flame-retardancy of thermosets was then assessed using PCFC (Table 2 and Figure 6B). DGEBA-based thermoset typically exhibits high peak heat release rate (pHRR) (529 W/g) at 377 °C, high total heat release (THR) and heat of complete combustion Δh (respectively 25.3 and 28 kJ/g). These values are similar to, but slightly lower than those obtained for DGEBA-IPDA thermosets [42]. The incorporation of phosphorus leads to a decrease in pHRR, THR, but also thermal stability. These tendencies are usual when phosphorus is present, especially phosphonate and phosphate groups which act as char promoters.

Interestingly HRR curves show two peaks for TECP material which may be ascribed to the heterogeneity of the network as already discussed. Another hypothesis is that the second peak corresponds to the decomposition of aliphatic chains. Indeed, the temperature of this peak is close to the decomposition temperature of polyolefins as polyethylene [43] or polypropylene. Moreover, this material does not exhibit additional charring and THR and Δh are higher compared to DGEBA thermoset. The decrease in pHRR is quite moderate. This may be assigned to the long aliphatic chains which are unable to char and contribute to heat release.

On the contrary TEEP, DEEP and DEEP-Ph lead to drastic decrease of pHRR and THR and strong enhancement of char. The heat of combustion is also lowered. Such high performances in comparison to TECP thermosets are due to the higher aromatic content of TEEP, DEEP and DEEP-Ph but also to the higher phosphorus content of corresponding thermosets. We observe that there is no significant difference between phosphate and phosphonate-containing materials. The DEEP-Ph thermoset exhibits a complex decomposition pathway and a very broad range of decomposition temperature (270–550 °C) allowing an important decrease of the heat release rate in comparison to other phosphate-containing thermosets. Char yields measured by PCFC are quite close to those obtained by TGA.

In order to get an insight on our results, PCFC data are compared to other comparable data already published. While sample size in cone calorimeter is not standard, it is not possible to compare properly our results.

Flammability of epoxy depends greatly on the groups present in the network. According to literature, THR can be expected in a large range from 11 to more than 30 kJ/g for phosphorus-free epoxy thermosets [44]. Note that in the work of Sonnier et al. a cardanol-containing epoxy thermoset exhibits a high THR and no char. This is in agreement with the present results showing that neither TECP, THR nor char content do not improve despite the presence of phosphorus.

Ménard et al. have compared the influence of phosphorus on different epoxy thermosets [45]. In this work, the phosphorus content does not exceed 3 wt.%. Phosphorus leads to a lower pHRR and THR and higher char content but the enhancement of flame retardancy is greatly dependent on the epoxy structure. The highest improvement is observed for a DGEBA thermoset while phosphorus does not reduce greatly the heat release at microscale for phloroglucinol-containing epoxy materials which are intrinsically less flammable. Nevertheless, the peak heat release rate is significantly reduced in all cases mainly because the decomposition becomes more complex in the presence of phosphorus and occurs within a large temperature range. Thermal stability is also reduced in presence of phosphorus, which is a very common observation.

Eugenol-based thermosets, and especially TEEP and DEEP ones, exhibit flammability data at microscale close to those obtained with a phloroglucinol-containing epoxy thermoset and difurfurylamine [45]: THR and pHRR are in the range 9–11 kJ/g and 80–120 W/g respectively depending of the phosphorus ratio (0–3 wt.%). But, in this case, the high level of flame retardancy is achieved for high phosphorus content (4.5–5.8 wt.%). Note also that TEEP-MXDA thermoset has flammability properties are very close to the TEEP-DDM thermoset studied by Miao et al. [28].

**Figure 7:**
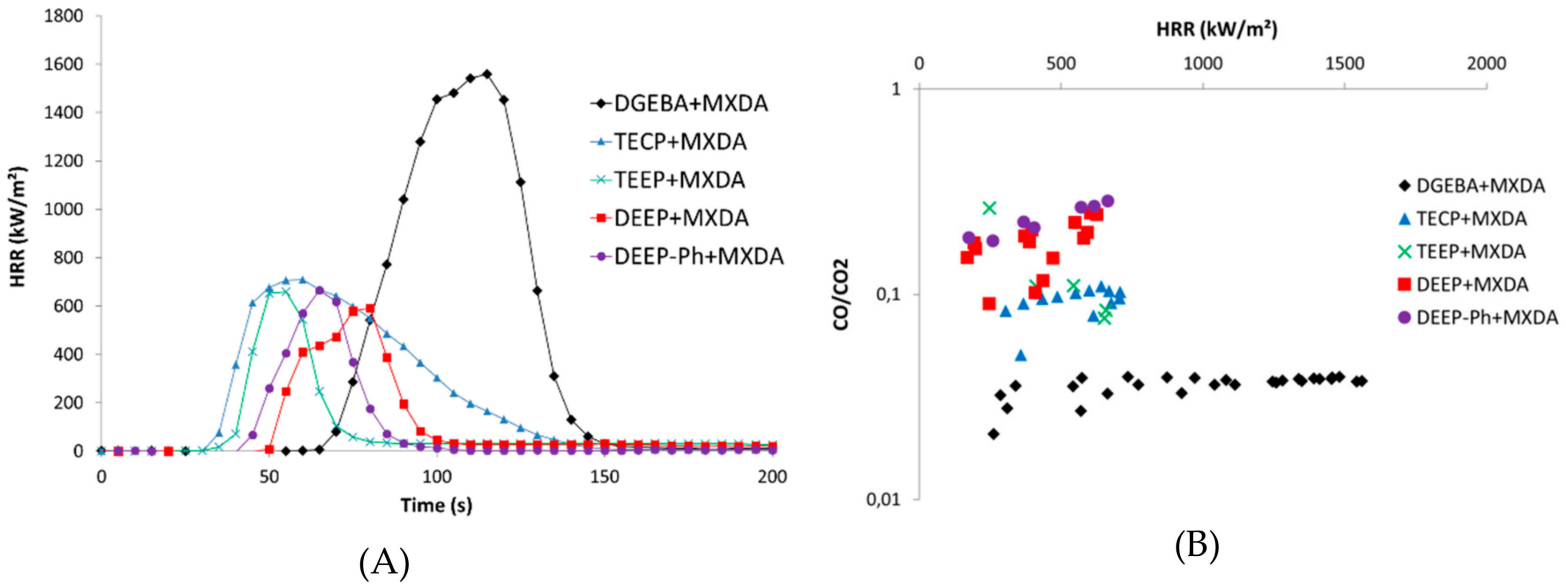
(**A**-left) Heat release rate versus time. (**B**-right) CO/CO_2_ ratio versus HRR in cone calorimeter tests.

Cone calorimeter tests were carried out to give an insight on the flammability at bench scale (Table 3 and Figure 7A). Indeed, some phenomena, as barrier effect or flame inhibition, are not effective in PCFC standard conditions. The comparison of phosphorus-containing thermosets with the DGEBA-MXDA thermoset is only qualitative because the initial mass of the latter is slightly higher. Moreover, some phenomena are less prominent for low-weight materials, as barrier effects due to char promotion. Indeed, such materials have not a fully thermally thick behavior. Nevertheless, the differences between them are so obvious as to draw meaningful conclusions.

The DGEBA-MXDA thermoset exhibits a very high pHRR (about 1500 kW/m^2^) and leaves only a small amount of char. Once again, this is similar to the fire behavior already observed for DGEBA-IPDA.

The incorporation of phosphorus leads to a significant decrease of flammability as evidenced by pHRR and THR values. Time-to-ignition is reduced with TECP, TEEP, DEEP and DEEP-Ph materials due to the decrease of thermal stability previously discussed. Once again, there are some significant differences between cardanol and eugenol-based thermosets. Actually, the phosphorylated eugenol containing thermosets possess intrinsically both lower pHRR and THR than the reference and the cardanol thermoset: they release less energy at lower release rate. The reasons for such results are discussed below.

From cone calorimeter results, the TECP thermoset shows a pHRR close to 700 kW/m^2^, a high THR (20.4 kJ/g) and moderate char content (15.4 wt.%). Interestingly, TECP and DGEBA thermosets have the same char content at microscale (10-12 wt.%) but a large difference is observed in Cone Calorimeter tests (15.4 wt.% and 2.3 wt.%, respectively). This is due to the presence of phosphorus which improves the thermo-oxidative stability of char avoiding its decomposition at the end of the test when the flame is vanished and pyrolysis becomes aerobic.

TEEP, DEEP and DEEP-Ph thermosets show a slightly lower pHRR (about 600 kW/m^2^) but a much lower THR (9–10 kJ/g) associated to enhanced charring (30–40 wt.%, slightly lower than values measured in TGA or PCFC).

**Figure 8:**
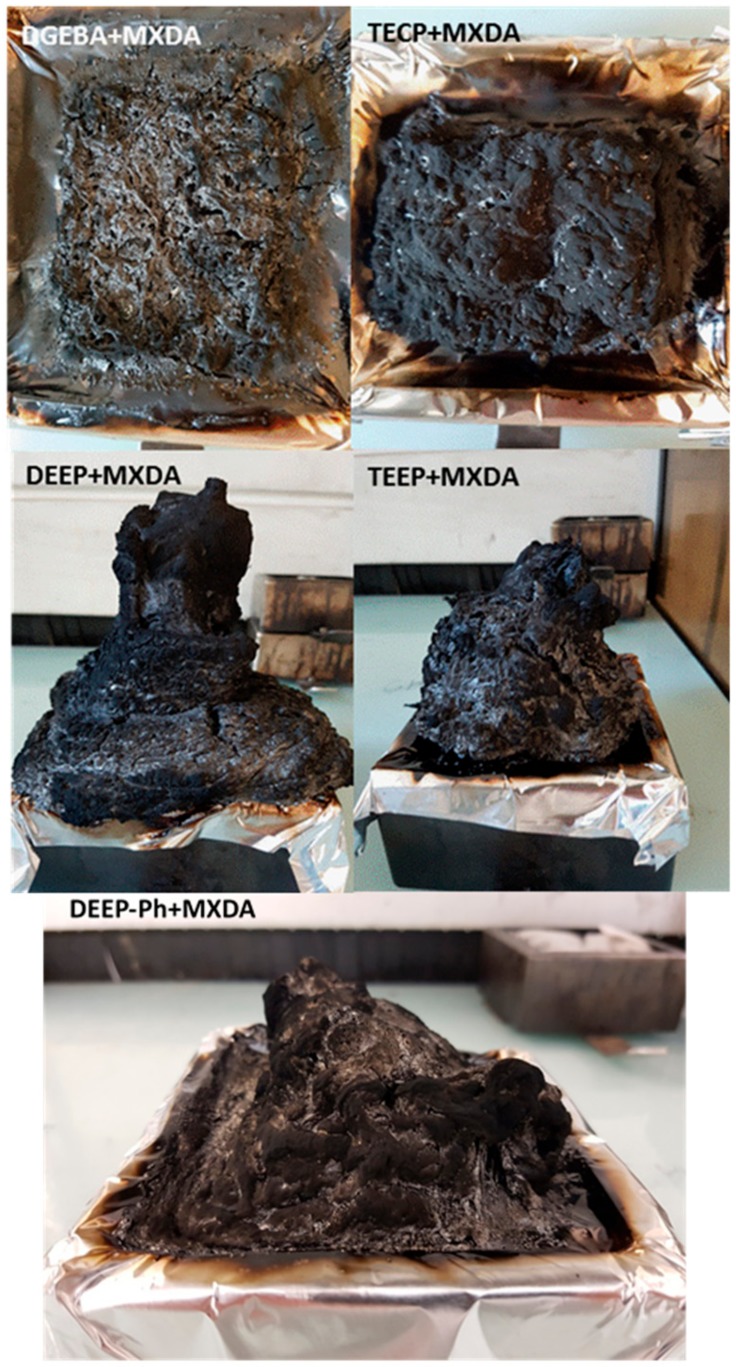
Pictures of residues from cone calorimeter tests.

Lastly, the residues are expanded for eugenol-containing epoxy materials, contrarily to residues from DGEBA and TECP thermosets (see Figure 8). The presence of the phosphorus function bonded to the epoxy network promotes the char as our team have already noted [42].

Undoubtedly, eugenol leads to better flame retardancy properties than cardanol. This must be assigned to the aliphatic chains of cardanol. These chains are surely unfavorable to char promotion and contain a high amount of carbon, contributing to heat release. But their presence also leads directly to a lower phosphorus content in the materials. As reported in Table 2 and Table 3, char content seems to roughly depend on phosphorus content.

As for PCFC, a slight difference is observed between TEEP, DEEP and DEEP-Ph thermosets. Phosphate and phosphonate groups are both equally efficient to promote char and reduce flammability. The influence of functionality (comparison between TEEP and DEEP materials) is not clear and may be counterbalanced by the higher phosphorus content of DEEP thermoset.

An interesting result concerns the influence of phosphorus on gas-phase mechanisms. Indeed, the effect heat of combustion (EHC) parameter highlighted the efficiency of the phosphorus radicals from phosphonate in the gas phase compared to the phosphate ones. Furthermore, while the char contents in the cone calorimeter and at a microscale (PCFC or TGA) are roughly similar, combustion efficiency Χ can be properly calculated as the ratio between EHC and the heat of complete combustion measured in PCFC. The combustion efficiency is around 0.8 for the DGEBA thermoset, which corresponds to quite well-ventilated combustion. Combustion efficiency decreases for phosphorus-containing thermosets, a little (almost non-significant) reduction was observed for DEEP and TEEP. The reduction is slightly more significant for DEEP and mostly DEEP-Ph (a 30% decrease was obtained for this last thermoset). This parameter X may be ascribed to the flame inhibition effect, which is quite uncommon for phosphate groups. Indeed, phosphorus with a high oxidation degree is known to act mainly or exclusively as a char promoter [29]. Alternately, Braun et al. explained that the influence of phosphonate on the inhibition flame was equivalent to phosphinate (less oxidation degree of phosphorus) [32], which corresponded to the phosphorus vaporization potential during pyrolysis. These results show the same trend with a good compromise of phosphonate function between actions in both gas and condensed phases.

To confirm a flame inhibition effect, CO/CO_2_ ratio was drawn versus heat release rate during intense burning (i.e., when HRR > 200 kW/m^2^) to avoid to take into account the oxidative decomposition of char at lower temperature at the end of the test (Figure 7B). The CO/CO_2_ ratio is very low for DGEBA thermoset but increases for phosphorus-containing materials. The highest values are reached for DEEP and DEEP-Ph. This confirms the incomplete combustion of these materials, and therefore the flame inhibition effect of phosphorus groups.

**Figure 9:**
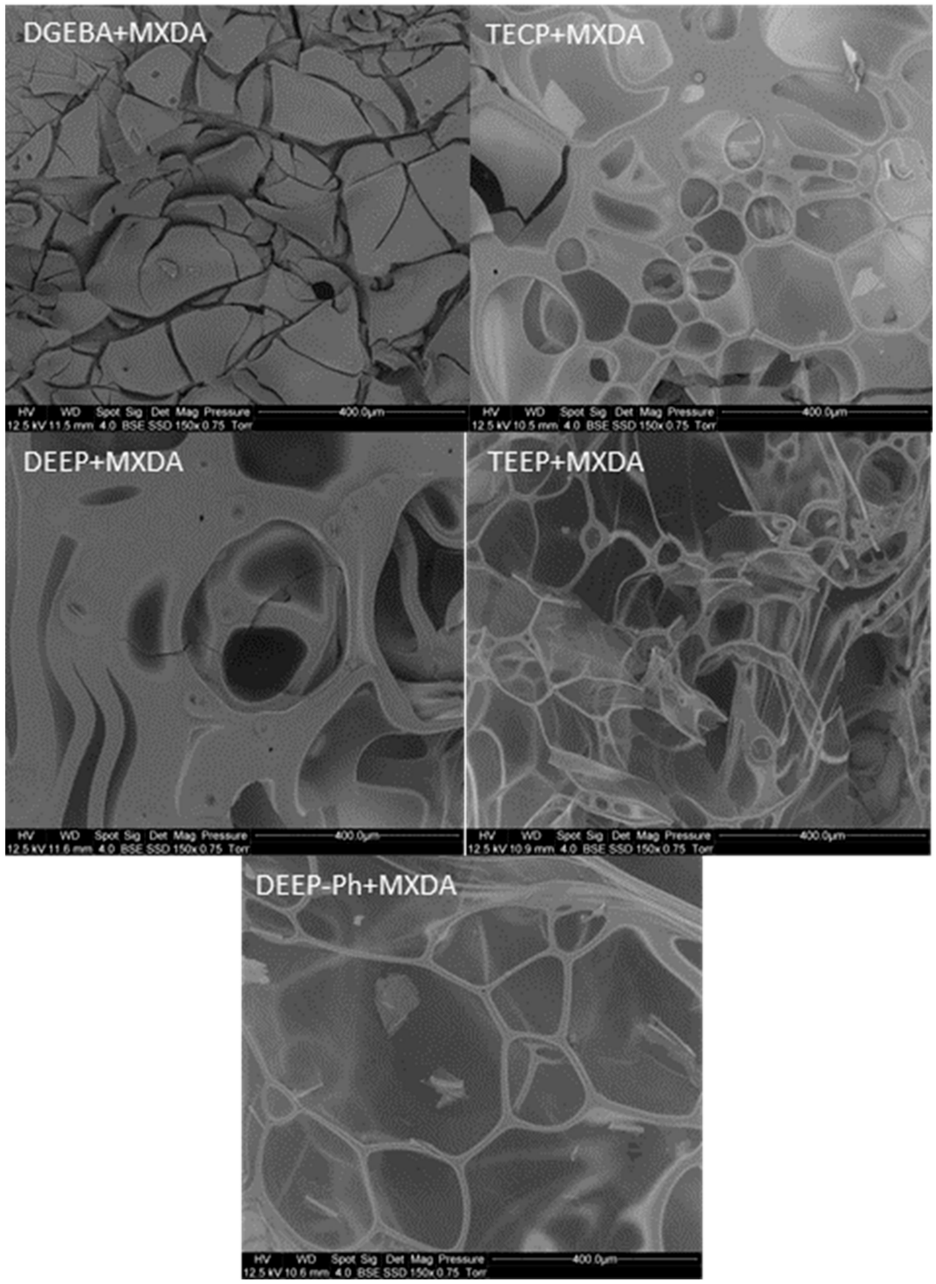
SEM pictures of residues from cone calorimeter.

Residues from cone calorimeter tests were analyzed in detail. Figure 9 shows SEM pictures of residues in bulk, except for DGEBA thermoset (because the residue is only a thin and non-expanded layer). In all other cases, residues are highly porous and appear as interconnected pores with thin walls. TEEP thermoset exhibits filamentous structures. Nevertheless, for TECP and especially DEEP thermosets, the walls are much thicker. Note that it is difficult to confirm these structures are fully representative for the whole residues due to their heterogeneous appearance.

Table 4 shows the elemental composition of these residues. A large sample was picked up, ground and analyzed in each case. The DGEBA reference shows the presence of carbon and oxygen. Carbon content decreases for phosphorus-containing thermosets and oxygen content increases. The phosphorus content is significantly different according to the residues. Residue from TECP, which contains initially only 2.4 wt.% of phosphorus exhibits the lowest content (8.7 wt.%). Nevertheless, residue from TEEP exhibits the highest content (11.7 wt.%) while TEEP thermoset has an intermediate phosphorus content (4.5 wt.%). Note that DEEP and DEEP-Ph have the same initial phosphorus content but residues do not show the same composition. This evidences that the composition does not depend only on the initial phosphorus content. It is also noteworthy that the oxygen content increases together with phosphorus content, as already observed in a previous work [46].

**Figure 10:**
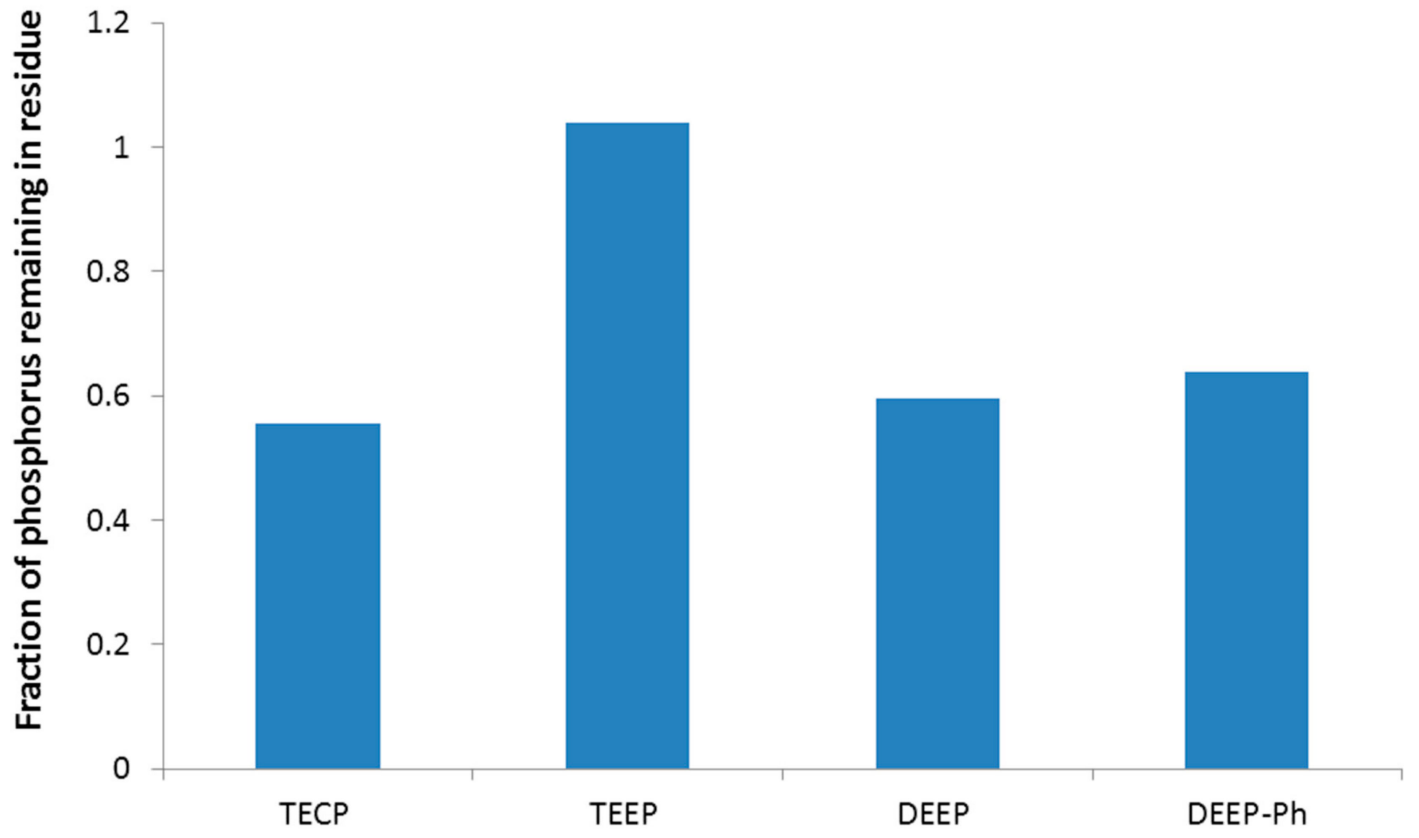
Fraction of phosphorus remaining in residue.

Considering the initial and final phosphorus contents (in the sample and in the residue) and the residue content, it is possible to calculate the fraction of phosphorus remaining in the residue after cone calorimeter. Figure 10 highlights that all the phosphorus content remains in condensed phase in the case of the thermoset based on TEEP. For the three other thermosets, the fraction of phosphorus remaining in condensed phase is close, between 0.55 and 0.65. This may be assigned to the trifunctionality of TEEP while DEEP and DEEP-Ph contain only two epoxy groups. For TECP, the long aliphatic chains of cardanol are detrimental and can explain why almost half of the phosphorus amount is released in the gas phase. Note also that the release of a significant fraction of phosphorus in gas phase is consistent with the flame inhibition effect previously proposed for DEEP and DEEP-Ph thermosets.

## 3. Experimental Part

### 3.1. Materials

Eugenol, phosphorus oxychloride, triethylamine, *meta*-chloroperoxybenzoic acid (*m*-CPBA) (77%), dichlorophenylphosphine oxide, phenyl dichlorophosphate, hydrogen peroxide (30% aqueous), formic acid and *p*-toluenesulfonic acid (PTSA) were purchased from Sigma Aldrich Merck (Darmstadt, Germany). Cardanol (Cardolite^®^ NX-2026) was purchased from Cardolite (Mariakerke, Belgium). *Meta*-xylylenediamine (MXDA) was obtained from CTP (Russelsheim, Germany). All materials were used as received. Deuterated solvent CDCl_3_ was obtained from Eurisotop for NMR study. Ethyl acetate (EtOAc), pentane and toluene were purchased from Sigma-Aldrich.

### 3.2. Methods

NMR analyses Proton and carbon Nuclear Magnetic Resonance (^1^H and ^31^P NMR) analyses were performed in deuterated chloroform (CDCl_3_) using a Bruker Avance 400 MHz NMR spectrometer at a temperature of 25 °C. NMR samples were prepared as follows: 10 mg of product for ^1^H experiment in around 0.4 mL of CDCl_3_. The chemical shifts were reported in part per million relative to tetramethylsilane. Spin multiplicity is shown by s = singlet, d = doublet, t = triplet, m = multiplet.

Thermogravimetric Analyses (TGA) were performed using a TG 209F1 apparatus (Netzsch) at a heating rate of 20 °C/min. Approximately 10 mg of sample was placed in a platinum crucible and heated from room temperature to 900 °C under nitrogen atmosphere (60 mL/min).

Differential Scanning Calorimetry (DSC) analyses were carried out using a NETZSCH DSC200F3 calorimeter. Constant calibration was performed using indium, *n*-octadecane and *n*-octane standards. Nitrogen was used as the purge gas. Approximately 10 mg of sample were placed in perforated aluminum pans and the thermal properties were recorded between −100 °C and 200 °C for the tri(epoxized-eugenyl)phosphate (TEEP) and tri(epoxized-cardanyl)phosphate (TECP)-based materials and from 25 °C to 250 °C for the di(epoxized-eugenyl)phosphonate (DEEP)-based material at 20 °C/min to observe the glass transition temperature. The T_g_ values were measured on the second heating ramp to erase the thermal history of the polymer. All the reported temperatures are mean values.

Dynamic mechanical analyses (DMA) were carried out on Metravib DMA 25 with Dynatest 6.8 software. Samples were tested according to uniaxial tension mode while heating at a rate of 3 °C/min from ≈ T_g_ − 100 °C to T_g_ + 100 °C, at a frequency of 1 Hz with a fixed strain of 10^−5^ m. These conditions have been chosen to study the elastic behavior of the materials. All the thermosets were analyzed at least two times for repeatability.

Titration of the epoxy equivalent weight by ^1^H NMR The Epoxy Equivalent Weight (EEW) is the amount of product needed for one equivalent of reactive epoxy function. It was determined by ^1^H NMR using an internal standard (benzophenone). A known weight of product and benzophenone was poured into an NMR tube and 500 μL of CDCl_3_ were added. It was determined using Equation (1) by comparing the integral of the protons of the benzophenone and the integral of the epoxy moiety.

(1) $$\mathrm{EEW}=\frac{\int {\mathrm{PhCOPh}\times H}_{\mathrm{epoxy}}\;}{\int {\mathrm{epoxy}\times H}_{\mathrm{PhCOPh}}}\;\times \;\frac{m_{\mathrm{epoxy}}}{m_{\mathrm{PhCOPh}}}{\times M}_{\mathrm{PhCOPh}},$$

where ∫_PhCOPh_ is the integration of the benzophenone protons; ∫_epoxy_ is the integration of the protons of the epoxy function; H_epoxy_ refers to the number of protons of the epoxy function; H_PhCOPh_ is the number of protons of the benzophenone; m_epoxy_ is the weight of the product; m_PhCOPh_ refers to the weight of the benzophenone, M_PhCOPh_ is the molecular weight of the benzophenone.

Cross-linking density from rubber elasticity theory [39,40], the uniaxial stretching was studied on the rubbery plateau at T > T_α_ + 50 (T = 150 °C for TEEP, DEEP and DGEBA and T = 100 °C for TECP), and at very small deformations. Under these hypotheses, the cross-linking density (ν′), can be obtained from Equation (2), where E′ is the storage modulus, R is gas constant and T_α_ is the temperature, in K, of the transition from vitreous to rubber domain of material determined at the maximum of the tan δ curve. Calculated values are given for information purposes only, and they can only be compared.

(2) $$\nu^{\prime }=\frac{E_{atT\alpha +50}^{\prime }}{3RT_{\alpha +50}},$$

Swelling index three samples of around 30 mg each were separately put in THF for 24 h. The swelling index (SI) was calculated using the Equation (3) where m_2_ is the mass of the material after swelling in THF and m_1_ is the initial mass of the material.

(3) $$\mathrm{SI}=\;\frac{m_{2}\;-{\;m}_{1}}{m_{1}}\times 100.$$

For the gel content after SI measurements, three samples were dried in a ventilated oven at 70 °C for 24 h. The gel content (GC) was calculated using the Equation (4), where m_3_ is the mass of the material after drying and m_1_ is the initial mass of the material.

(4) $$\mathrm{GC}=\;\frac{{\;m}_{3}}{m_{1}}\times 100,$$

Pyrolysis combustion flow calorimeter flammability was first investigated using a pyrolysis combustion flow calorimeter which was developed by Lyon and Walters [47]. The sample (3 ± 0.5 mg) was first heated from 80 to 750 °C at 1 °C/s in a pyrolyzer under nitrogen flow and the degradation products were sent to a combustor where they were mixed with oxygen in excess at 900 °C. In such conditions, these products were fully oxidized. The heat release rate (HRR) was then calculated by oxygen depletion according to Huggett’s relation (1 kg of consumed oxygen corresponds to 13.1 MJ of released energy) [48].

Cone calorimeter fire behavior was also studied using a cone calorimeter (fire testing technology) which is a powerful tool to investigate the fire behavior of polymers at bench scale. Samples were previously conditioned away from air and moisture. A horizontal sample sheet of 70 × 80 × 2 mm^3^ was placed at 2.5 cm below a conic heater and isolated by rock wool. Plates were wrapped in aluminum foil but no retained frame was used. The samples were exposed to a heat flux of 35 kW/m^2^ in well-ventilated conditions (air rate 24 L/s) in the presence of a spark igniter to force the ignition. HRR was determined according to oxygen depletion (Huggett’s relation) as in the pyrolysis combustion flow calorimeter (PCFC). Note that the dimensions of samples are not standard and their masses are quite low. The standard deviations of cone calorimeter measurements are around 15%.

Scanning electron microscopy coupled with energy-dispersive X-ray spectroscopy (SEM-EDX). The bulk structure of residues was observed using an SEM microscope (FEI Quanta 200 SEM). Elemental composition was analyzed with energy-dispersive X-ray detector (Oxford) coupled with the SEM microscope. For each residue, three analyses were carried out. Note that in the case of reference epoxy, residue is not expanded and only the surface was analyzed.

### 3.3. Monomers/Polymers Syntheses

#### 3.3.1. Synthesis of Trieugenylphosphate (TEP)

The synthesis of TEP is described as follows: Eugenol (10 g, 61 mmol) and triethylamine (6.2 g, 61 mmol) was solubilized in ethyl acetate (100 mL). Phosphorus oxychloride (3.12 g, 20 mmol) was added dropwise, at 0 °C, to the solution. The reaction mixture was stirred at room temperature for 24 h. When the reaction was complete, the mixture was diluted with 100 mL of ethyl acetate and washed three times with 100 mL of deionized (DI) water and one time with brine solution (100 mL). The organic layer was dried with anhydrous magnesium sulfate and filtered. The filtrate was purified by silica gel chromatography (8:2 Pentane:EtOAc) to give 10.6 g of TEP as yellow oil (97% yield).

^1^H NMR (400 MHz, CDCl_3_): δ (ppm) 7.31 (1H, dd, *J* = 1.4 and 8.2 Hz); 6.67–6.76 (2H, m); 5.87–5.99 (1H, m); 5.03–5.14 (2H, m); 3.75 (3H, s); 3.34 (2H, d, *J* = 6.7 Hz).

^31^P NMR (162 MHz, CDCl_3_, TMS): −15.78.

#### 3.3.2. Synthesis of Tricardanylphosphate (TCP)

The synthesis of TCP is described previously with: cardanol (50 g, 166 mmol), triethylamine (22.4 mL, 166 mmol), ethyl acetate (410 mL) and phosphorus oxychloride (15.3 mL, 55 mmol). Purification: silica gel chromatography (9:1 Pentane:EtOAc) to give 40 g of TCP as yellow oil (73% yield).

^1^H NMR (400 MHz, CDCl_3_): δ (ppm) 7.21–7.25 (1H, m); 6.98–7.08 (3H, m); 5.76–5.88 (0.46H, m); 5.27–5.49 (4.1H, m); 4.94–5.10 (1H, m); 2.75–2.88 (2.4H, m); 2.54–2.63 (2.4H, m); 1.94–2.12 (4.4H, m); 1.52–1.66 (2.5H, m); 1.16–1.45 (17.4H, m); 0.84–0.96 (2.7H, m).

^31^P NMR (162 MHz, CDCl_3_, TMS): −17.66.

#### 3.3.3. Synthesis of Dieugenylphosphate (DEP)

The synthesis of DEP is described previously with: eugenol (50 g, 305 mmol), triethylamine (41 mL, 305 mmol), ethyl acetate (750 mL) and phenyl dichlorophosphate (22 mL, 152 mmol). Purification: silica gel chromatography (8:2 Pentane:EtOAc) to give 16 g of DEP as yellow oil (24% yield).

^1^H NMR (400 MHz, CDCl_3_): δ (ppm) 7.33–7.39 (4H, m); 7.17–7.25 (3H, m); 6.68–6.82 (4H, m); 5.90–6.00 (2H, m); 5.04–5.16 (4H, m); 3.76 (6H, s); 3.37 (4H, d, *J* = 6.9 Hz).

^31^P NMR (162 MHz, CDCl_3_, TMS): −12.82.

#### 3.3.4. Synthesis of Dieugenylphosphonate (DEP-Ph)

The synthesis of DEP-Ph is described previously with: eugenol (34 g, 207 mmol), triethylamine (28 mL, 207 mmol), ethyl acetate (512 mL) and phenylphosphonic dichloride (20 mL, 103 mmol). Purification: silica gel chromatography (8:2 Pentane:EtOAc) to give 24 g of DEP-Ph as a white solid (52% yield).

^1^H NMR (400 MHz, CDCl_3_): δ (ppm) 8.0–8.10 (2H, m); 7.42–7.59 (3H, m); 7.17 (2H, dd, *J* = 1.7 and 8.2 Hz); 6.64–6.72 (4H, m); 5.84–5.99 (2H, m); 4.98–5.10 (4H, m); 3.68 (6H, s); 3.32 (4H, d, *J* = 6.7 Hz).

^31^P NMR (162 MHz, CDCl_3_, TMS): 12.94.

#### 3.3.5. Synthesis of Tri(epoxized-eugenyl)phosphate (TEEP)

TEEP was synthesized as follows. A solution of TEP (10.2 g, 19 mmol) in ethyl acetate (50 mL) was added dropwise to a cooled suspension of *meta*-chloroperbenzoïc acid (m-CPBA 77%) (14 g, 62.7 mmol) in ethyl acetate (200 mL). The reaction mixture was stirred at room temperature for 24 h. The organic layer was washed several times with saturated sodium bicarbonate, brine and deionized water. The organic layer was dried with anhydrous magnesium sulfate and filtered. The filtrate was distilled under vacuum and purified by column chromatography (Pentane/EtOAc 50:50) to afford 10.3 g of TEEP as brown viscous oil (93% yield).

^1^H NMR (400 MHz, CDCl_3_, TMS): δ (ppm) 7.30–7.37 (1H, m); 6.73–6.87 (2H, m); 3.78 (3H, s); 3.10–3.16 (1H, m); 2.75–2.96 (3H, m); 2.53 (1H, dd, *J* = 2.7 and 4.9 Hz).

^31^P NMR (162 MHz, CDCl_3_, TMS): −15.92.

#### 3.3.6. Synthesis of Tri(epoxized-cardanyl)phosphate (TECP)

TECP was synthesized as follows: A solution of TCP (10 g, 10.5 mmol), formic acid (360 μL, 9.5 mmol), *p*-toluenesulfonic acid (180 mg, 0.38 mmol) in toluene or EtOAc (4 mL) was heated to 50 °C. Then, a hydrogen peroxide solution (30% (*w/w*) in H_2_O) (11 g, 32.3 mmol) was added dropwise. The reaction mixture was stirred at 65 °C for 24 h. The organic layer was washed several times with saturated sodium bicarbonate, brine and deionized water. The organic layer was dried with anhydrous magnesium sulfate and filtered. The filtrate was distilled under vacuum and purified by column chromatography (Pentane/EtOAc 8:2).

^1^H NMR (400 MHz, CDCl_3_): δ (ppm) 7.18–7.25 (1H, m); 6.97–7.06 (3H, m); 5.74–5.94 (0.30H, m); 5.4–5.62 (0.29H, m); 4.95–5.22 (0.56H, m); 3.03–3.21 (0.80H, m); 2.86–3.01 (1.58H, m); 2.57 (2H, t, *J* = 7.8 Hz); 2.14–2.45 (0.83H, m); 1.69–1.85 (0.68H, m); 1.18–1.67 (18.1H, m); 0.82–1.03 (2.03H, m).

^31^P NMR (162 MHz, CDCl_3_, TMS): −17.65.

#### 3.3.7. Synthesis of Di(epoxized-eugenyl)phosphate (DEEP)

The synthesis of DEEP is described previously with: DEP (16 g, 35 mmol), *m*-CPBA (16 g, 70 mmol) and ethyl acetate (430 mL). Purification: silica gel chromatography (2:8 Pentane:EtOAc) to give 11 g of DEEP as yellow oil (65% yield).

^1^H NMR (400 MHz, CDCl_3_): δ (ppm) 7.35–7.39 (4H, m); 7.19–7.27 (3H, m); 6.77–6.88 (4H, m); 3.79 (6H, s); 3.13–3.19 (2H, m); 2.80–2.89 (6H, m); 2.56 (2H, dd, *J* = 2.7 and 5.0 Hz).

^31^P NMR (162 MHz, CDCl_3_, TMS): −16.52.

#### 3.3.8. Synthesis of Di(epoxized-eugenyl)phosphonate (DEEP-Ph)

The synthesis of DEEP-Ph is described previously with: DEP-Ph (24 g, 53 mmol), *m*-CPBA (29.5 g, 117 mmol) and ethyl acetate (680 mL). Purification: silica gel chromatography (2:8 Pentane:EtOAc) to give 14 g of DEEP-Ph as a white solid (55% yield).

^1^H NMR (400 MHz, CDCl_3_): δ (ppm) 8.04–8.12 (2H, m), 7.48–7.64 (3H, m); 7.21 (2H, dd, *J* = 1.5 and 8.0 Hz); 6.72–6.83 (4H, m); 3.75 (6H, s); 3.12–3.18 (2H, m); 2.78–2.86 (6H, m); 2.55 (2H, ddd, *J* = 0.8, 2.6 and 5.0 Hz).

^31^P NMR (162 MHz, CDCl_3_, TMS): 13.00.

#### 3.3.9. Epoxy Network Curing Protocol

Several formulations were carried out using TEEP, TECP, DEEP, DEEP-Ph, DGEBA and MXDA. In order to prepare the materials, the mass of amine was calculated using the Equation (5). The Amine Hydrogen Equivalent Weight (AHEW) and the Epoxy Equivalent Weight represent the amount of product needed for one equivalent of reactive function.

(5) $$m_{amine\;}=\;\frac{AHEW_{amine}\;\times \;m_{epoxy}}{EEW},$$

The materials were synthesized with a molar ratio epoxy/amine of 2:1. Indeed, two epoxy groups react with each hydrogen of the primary amine. The amine and the epoxy were stirred using a speedmixer under vacuum and then the homogeneous mixture obtained was put in an aluminum mold for three hours at 80 °C and then cured at 150 °C for one hour for the eugenol derivatives, while for the cardanol derivative the mixture was heated at 120 °C overnight and then cured at 150 °C for three hours. Sheets of 7 × 8 cm^2^ have been synthetized to characterize flame retardant properties.

## 4. Conclusions

In this paper, we synthesized four biobased epoxy monomers from cardanol and eugenol without using BPA or epichlorohydrin. Four different thermosets have been obtained after curing with a benzylic amine (MXDA) and their thermo-mechanical properties have been compared to a petroleum-based epoxy thermoset coming from DGEBA. Due to the high aromatic density of eugenol-based materials, high glass transition temperatures are obtained whereas the long aliphatic chain of cardanol monomers leads to flexible thermosets with low T_g_. These new eugenol-based thermosets exhibit good thermo-mechanical properties with high char yields. The thermal stability of these thermosets was finally characterized by TGA and their flame retardant properties by PCFC and cone calorimeter tests. The incorporation of phosphorus as a linker between phenols confers flame-retardant properties to the networks, without the use of flame-retardant additives. This reactive approach prevents the migration of flame-retardant additives out of the network. Furthermore, phosphonate or phosphate functions do not hang off the main chain of the network and are completely integrated to it. Hence, the incorporation of phosphorus leads to a significant decrease of flammability as evidenced by pHRR and THR values. Furthermore, eugenol lead to monomers with better flame retardant properties than cardanol which contains long aliphatic chain. This chain is unfavorable to the char promotion and contains high carbon amount, contributing to heat release. Furthermore, phosphate and phosphonate groups are both equally efficient to promote char and reduce flammability for these materials. Moreover the residues are expanded, contrarily to residues from DGEBA and TECP thermosets, relating to great intumescence properties. The flame inhibition effect was confirmed as an additional flame retardancy mode-of-action with a better efficiency for the phosphonate function. In conclusion, novel biobased aromatic monomers were synthesized, giving access to high performance thermosets with good thermal, thermo-mechanical and flame retardant properties.

## Figures and Tables

**Table 1:** Thermal and thermo-mechanical properties of cured materials.

Epoxy Monomers (Cured With MXDA)	Gel Content (%)	T_d5%_ (°C)	Char Yield (%) ^a^	T_g_ (°C)	T_α_ (°C)	E′_glassy_ (MPa)	E′_rubbery_ (MPa)	ν′ (mol/m^3^)
DGEBA	99	333	7	116	130	1210	18	1146
TECP	82	311	12	−26	18	1500	1.3	140
TEEP	99	295	43	84	78	1210	2.5	218
DEEP	100	300	41	98	94	2000	7.8	739
DEEP-Ph	100	305	35	109	128	1250	3.1	273

^a^ at 900 °C under nitrogen.

**Table 2:** Main data from pyrolysis-combustion flow calorimeter tests.

Epoxy Monomers (Cured with MXDA)	P Content (wt.%)	pHRR1 (W/g)	TpHRR1 (°C)	pHRR2 (W/g)	TpHRR2 (°C)	THR (kJ/g)	Residue Content (wt.%)	Δh (kJ/g)
DGEBA	0	529	377			25.3	11	28.3
TECP	2.4	137	314	292	463	29.5	12	33.4
TEEP	4.5	95	329			9.6	55	21.1
DEEP	5.8	117	338			11.5	48	21.9
DEEP-Ph	5.8	88	366			15.3	35	23.6

**Table 3:** Main data from cone calorimeter tests.

Epoxy Monomers (Cured with MXDA)	P Content (wt.%)	TTI (s)	pHRR (kW/m^2^)	THR (kJ/g)	Residue Content (wt.%)	EHC (kJ/g)	ΔResidue (%) *	Χ
DGEBA	0	65	1486	22.1	2.3	22.7	−4.7	0.80
TECP	2.4	31	708	20.4	15.4	24.1	3.4	0.72
TEEP	4.5	38	659	9.5	40	15.8	−3	0.75
DEEP	5.8	39	593	9.3	37.5	14.9	−3.5	0.68
DEEP-Ph	5.8	40	665	8.4	32.8	12.5	−2.2	0.53

* ΔResidue = % residue in cone − % residue in TGA.

**Table 4:** Elemental composition of residues.

	C (wt.%)	O (wt.%)	P (wt.%)
DGEBA	82.1 (±0.9)	17.3 (±0.6)	/
TECP	66.1 (±0.4)	24.3 (±0.1)	8.7 (±0.2)
TEEP	58.3 (±0.2)	29.6 (±0.1)	11.7 (±0.1)
DEEP	68.1 (±0.1)	22.4 (±0.3)	9.2 (±0.0)
DEEP-Ph	62.0 (±0.1)	26.1 (±0.0)	11.3 (±0.2)

## Supplementary Materials

The following are available online, Figures S1–S12.
