# Supplementary material for: Cardanol and Eugenol Based Flame Retardant Epoxy Monomers for Thermostable Networks
Source: Molecules. 2019 May 10;24(9):1818. doi: 10.3390/molecules24091818 (PMC6540237; doi:10.3390/molecules24091818)
Supplement: Supplementary file 1 [file molecules-24-01818-s001.pdf]

CARDANOL AND EUGENOL BASED FLAME RETARDANT EPOXY MONOMERS FOR THERMOSTABLE NETWORKS

Yvan Ecochard,[a†] Mélanie Decostanzi,[a†] Claire Negrell,[a] Rodolphe Sonnier,[b] Sylvain Caillol[a*]

[a] ICGM, UMR 5253 – CNRS, Université de Montpellier, ENSCM, 240 Avenue Emile Jeanbrau 34296 Montpellier, France

[b] C2MA, IMT – Mines Alès, 6, avenue de Clavières, 30100 Alès, France

[†] all authors have equally contributed

*Corresponding author: Sylvain Caillol, Email: sylvain.caillol@enscm.fr

Content

I. NMR analyses 2

1. TEP 2
2. TEEP 3
3. DEP 4
4. DEEP 5
5. DEP-Ph 6
6. DEEP-Ph 7
7. TCP 8
8. TECP 9

II. DSC analyses 10

I.NMR analyses

1. TEP

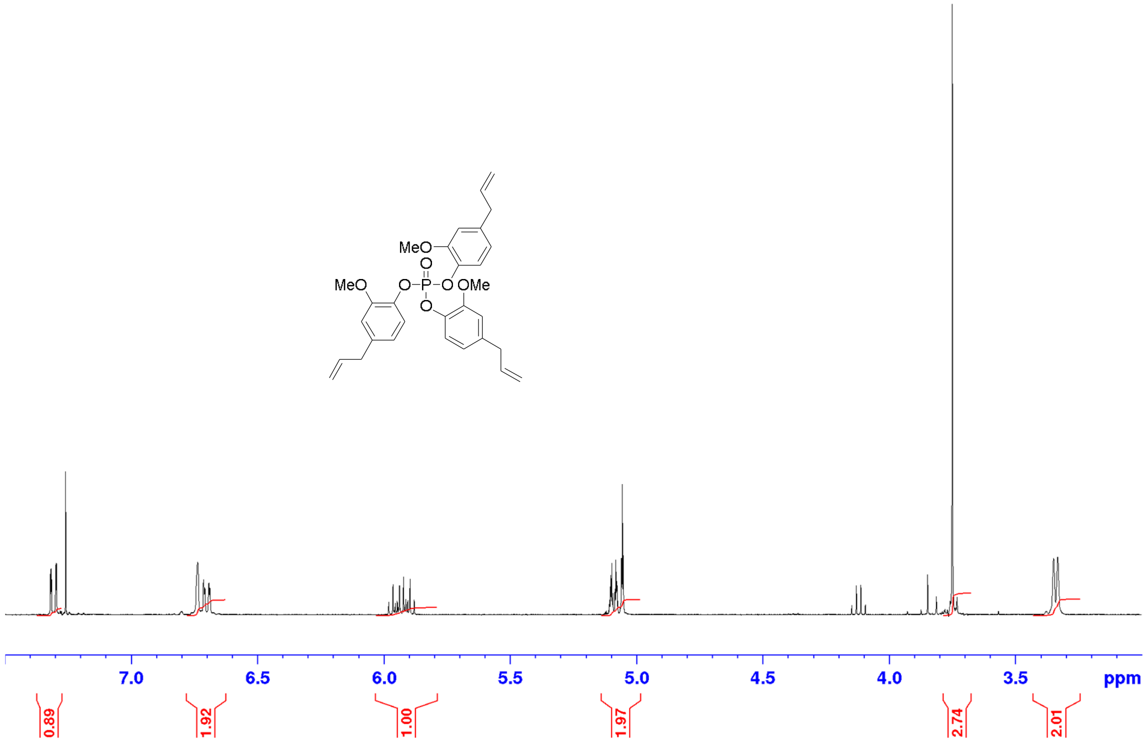

Figure S1: ^{1}H NMR of TEP.

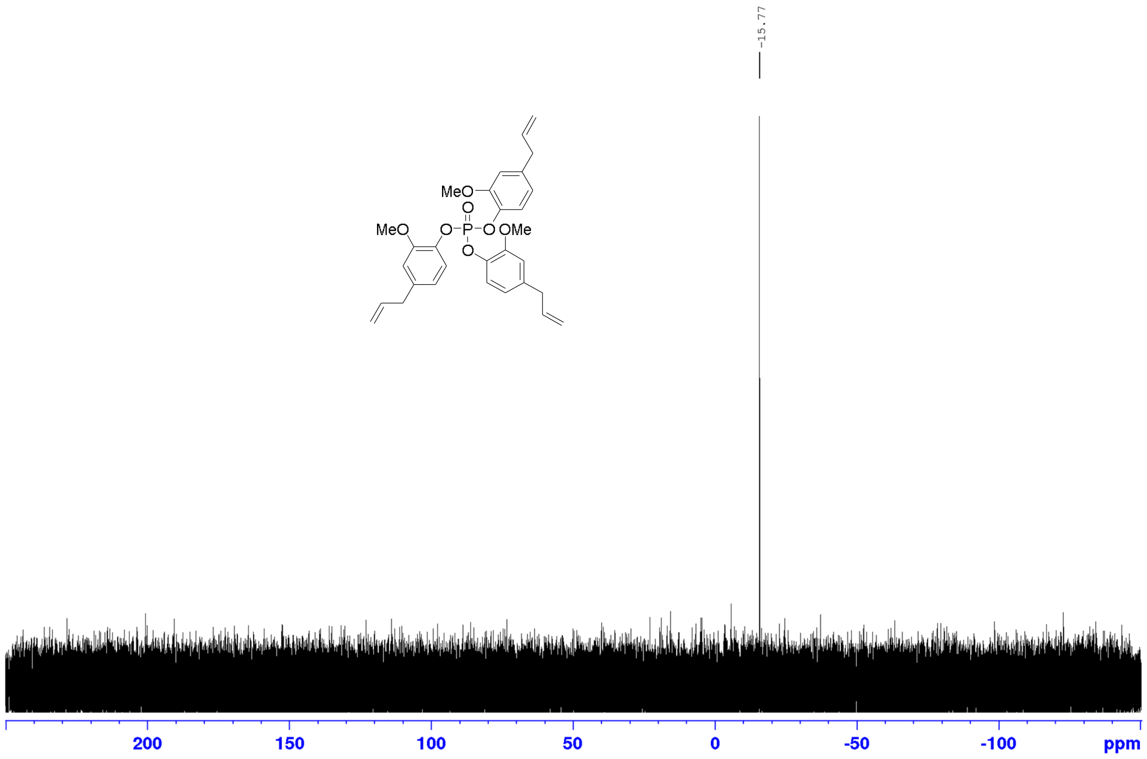

Figure S2: ^{31}P NMR of TEP.

2. <u>TEEP</u>

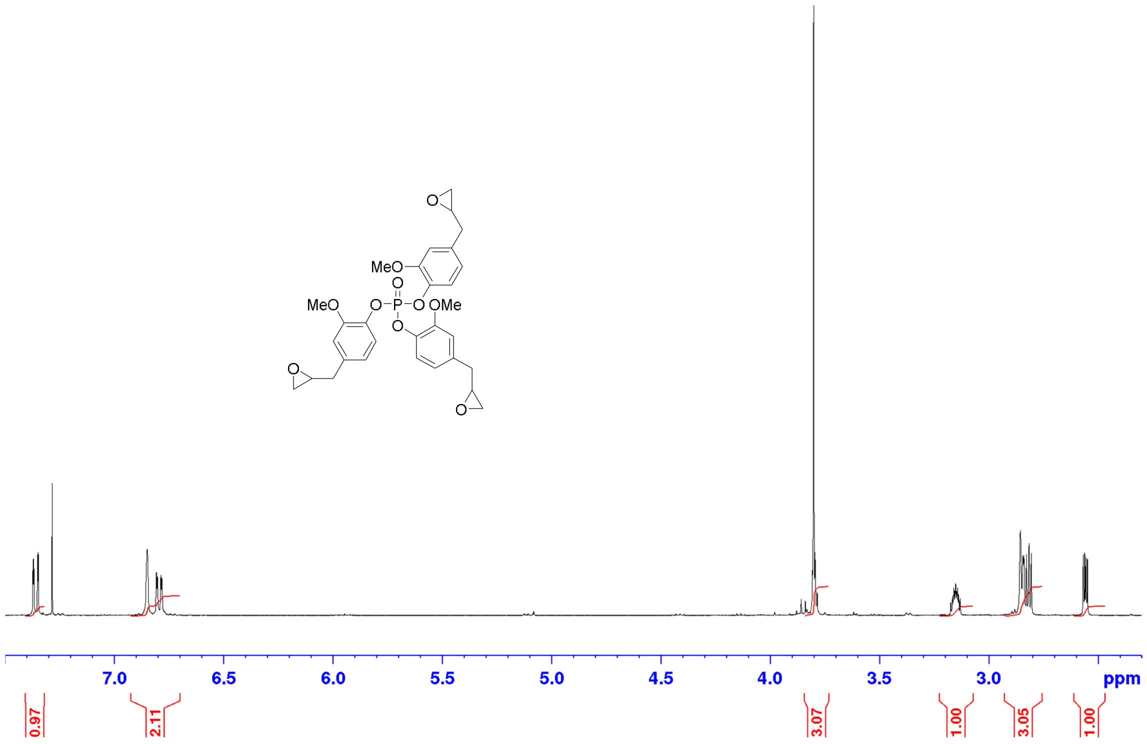

Figure S3: ^{1}H NMR of TEEP.

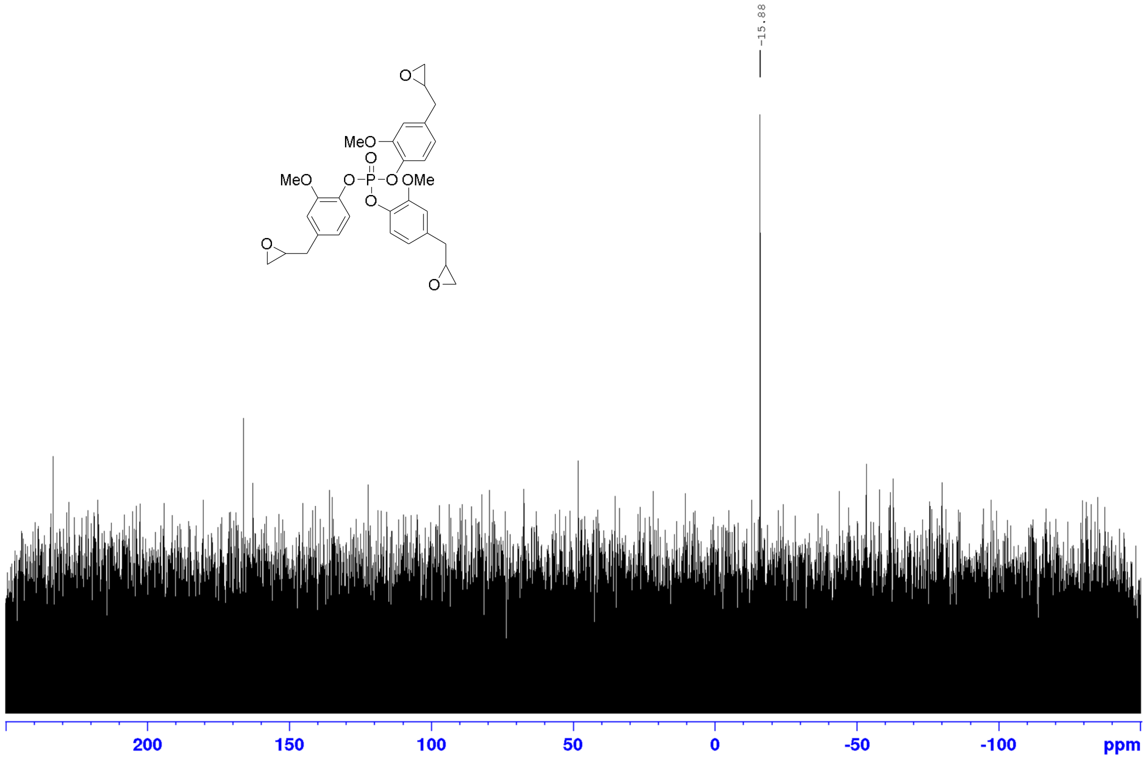

Figure S4: ^{31}P NMR of TEEP.

3. DEP

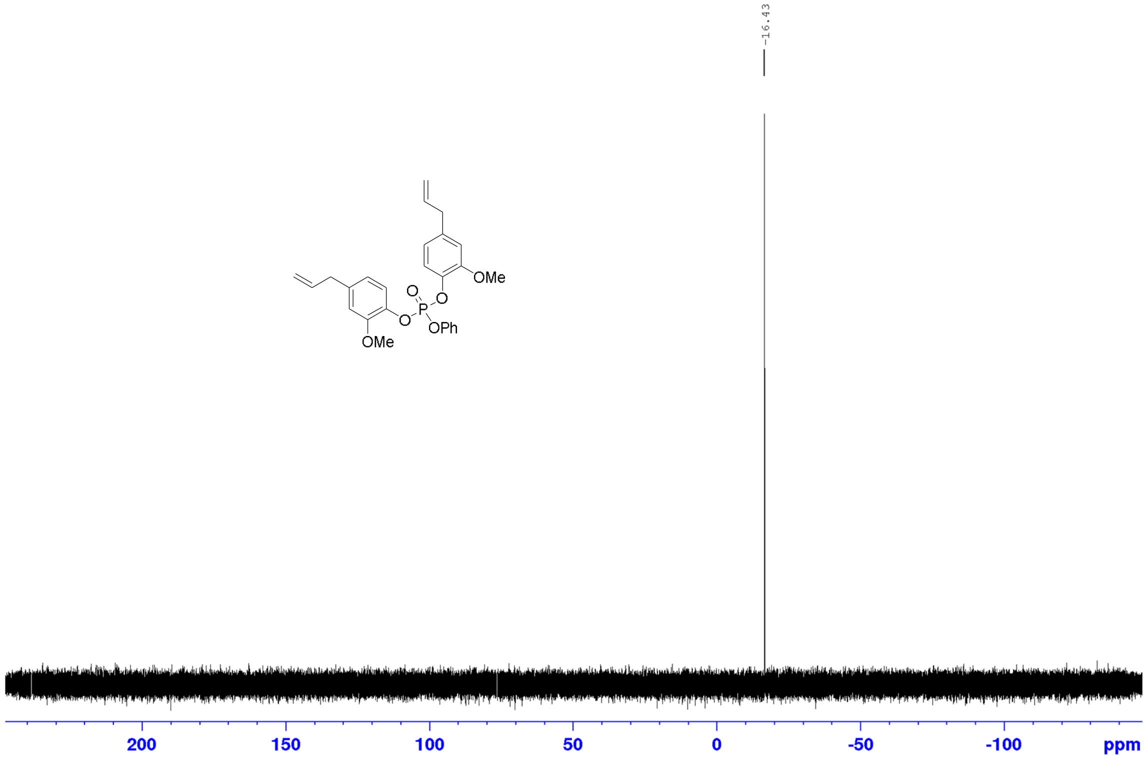

Figure S5: ^{31}P NMR of DEP.

4. DEEP

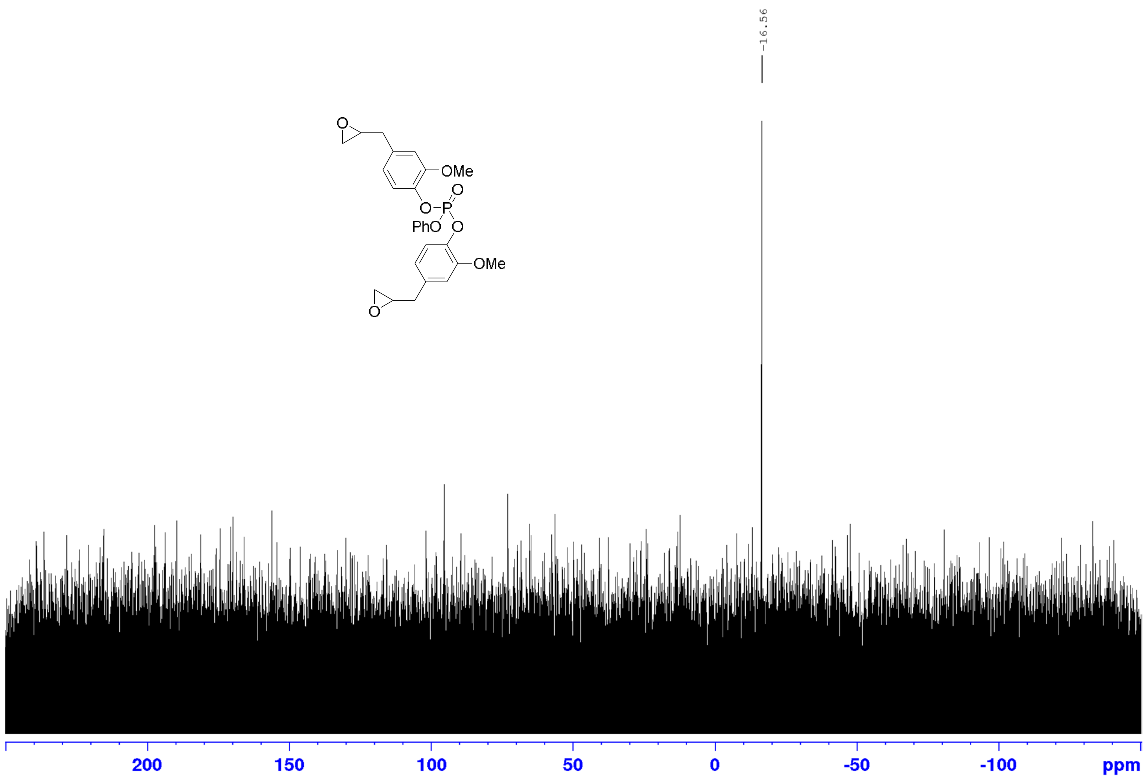

Figure S6: ³¹P NMR of DEEP.

5. DEP-Ph

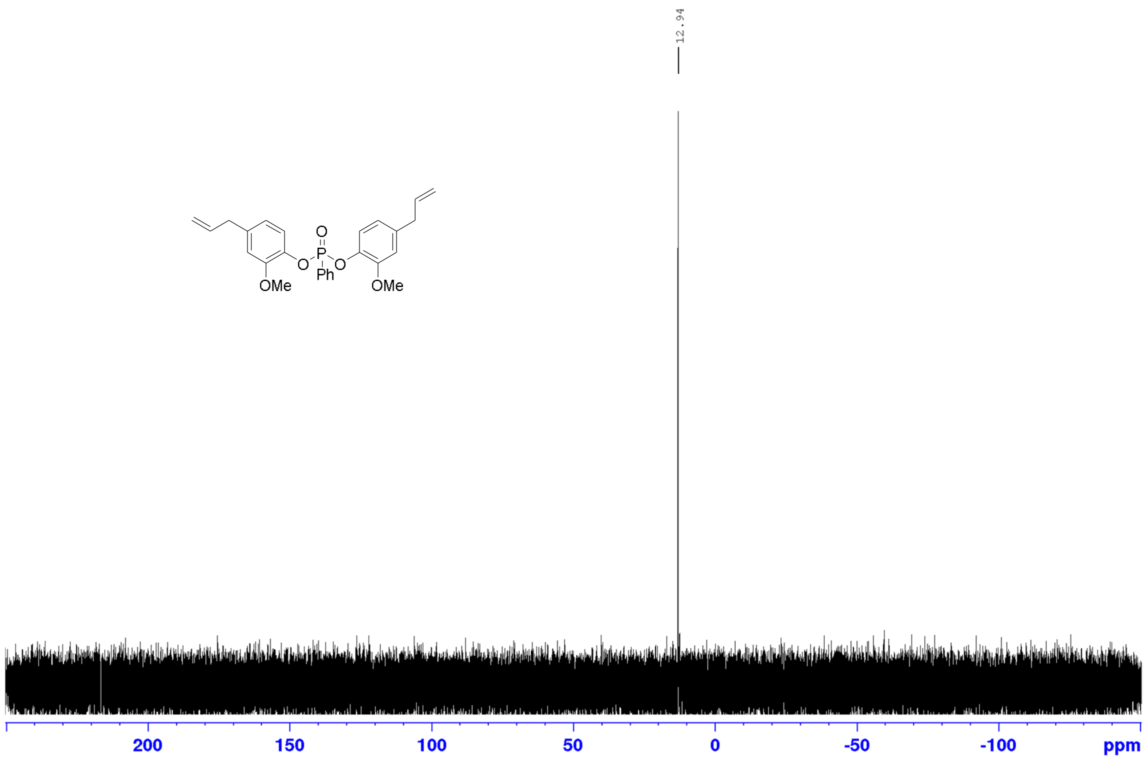

Figure S7: ^{31}P NMR of DEP-Ph.

6. <u>DEEP-Ph</u>

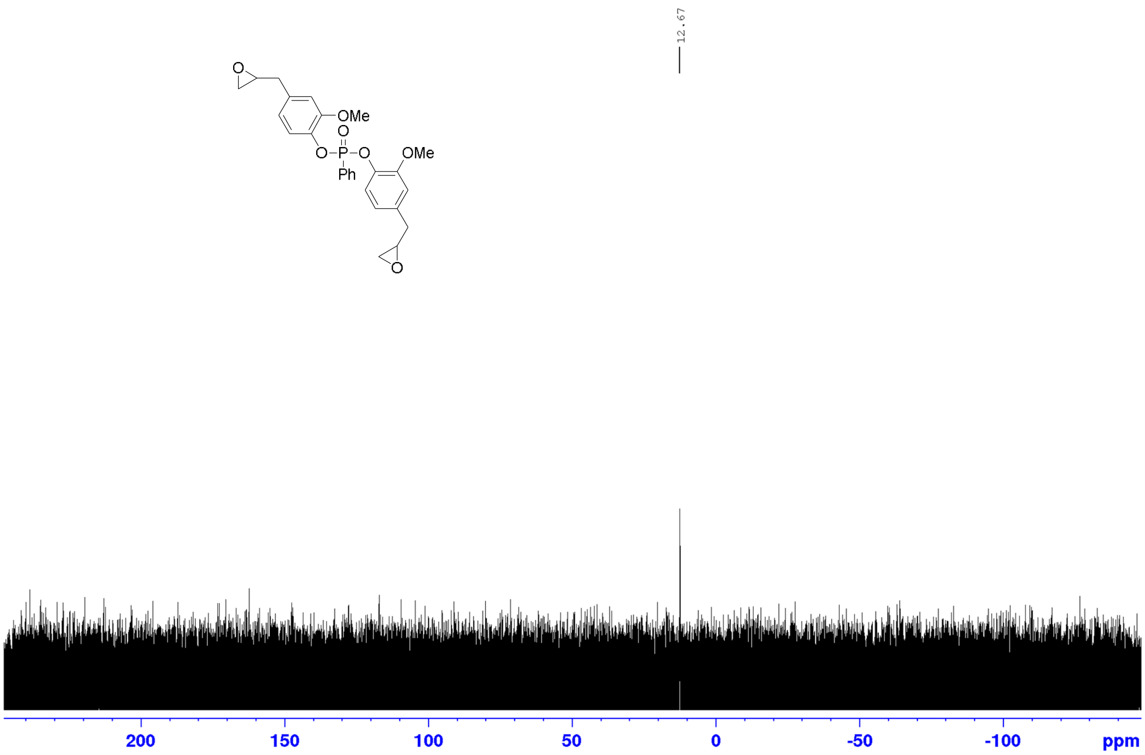

Figure S8: ^{31}P NMR of DEEP-Ph.

7. TCP

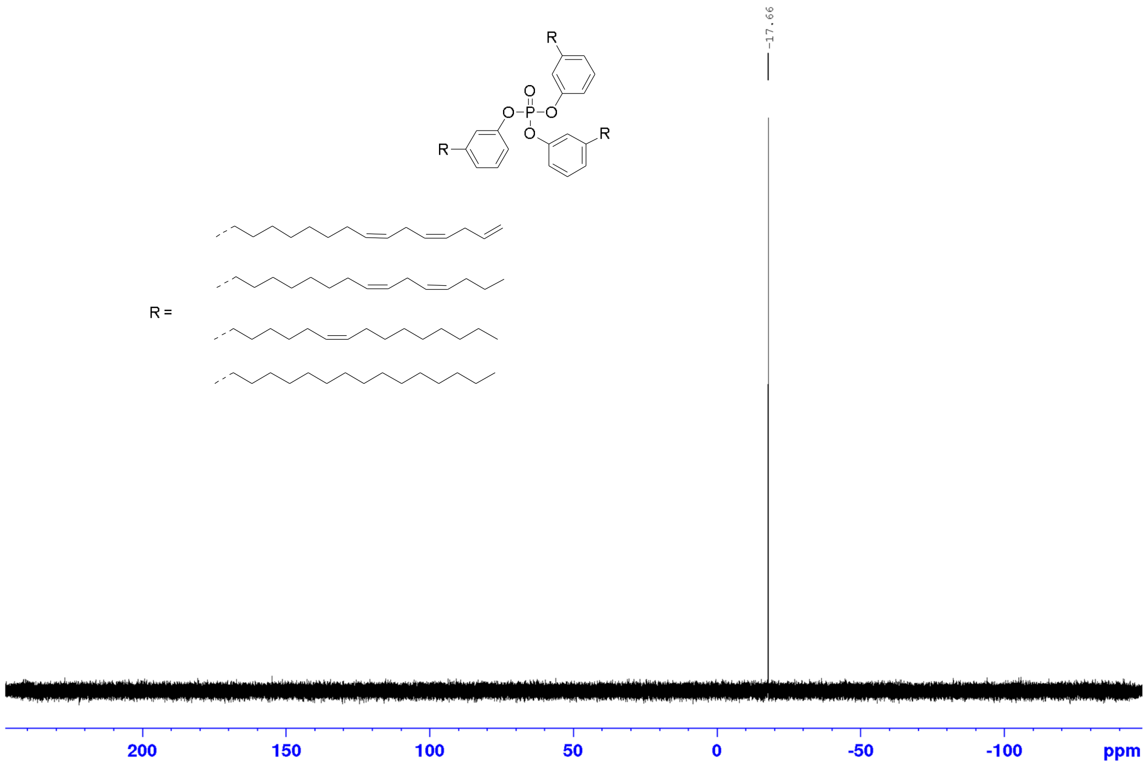

Figure S9: ^{31}P NMR of TCP.

8. TECP

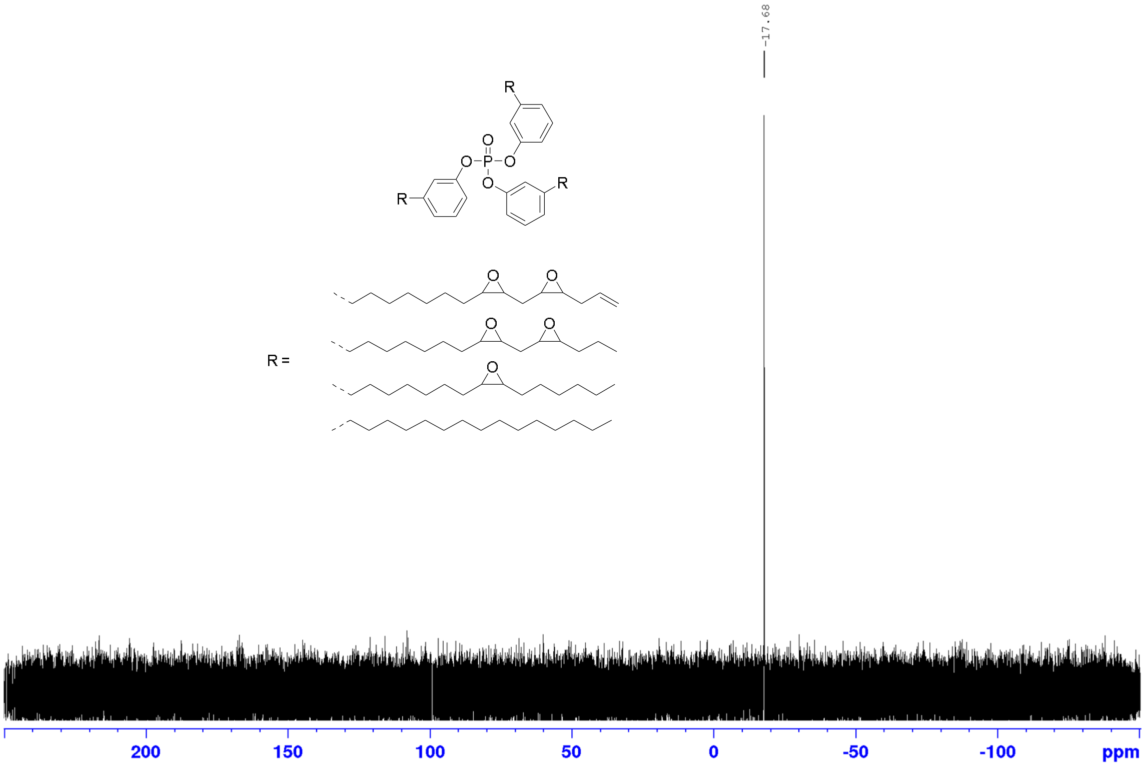

Figure S10: ^{31}P NMR of TECP.

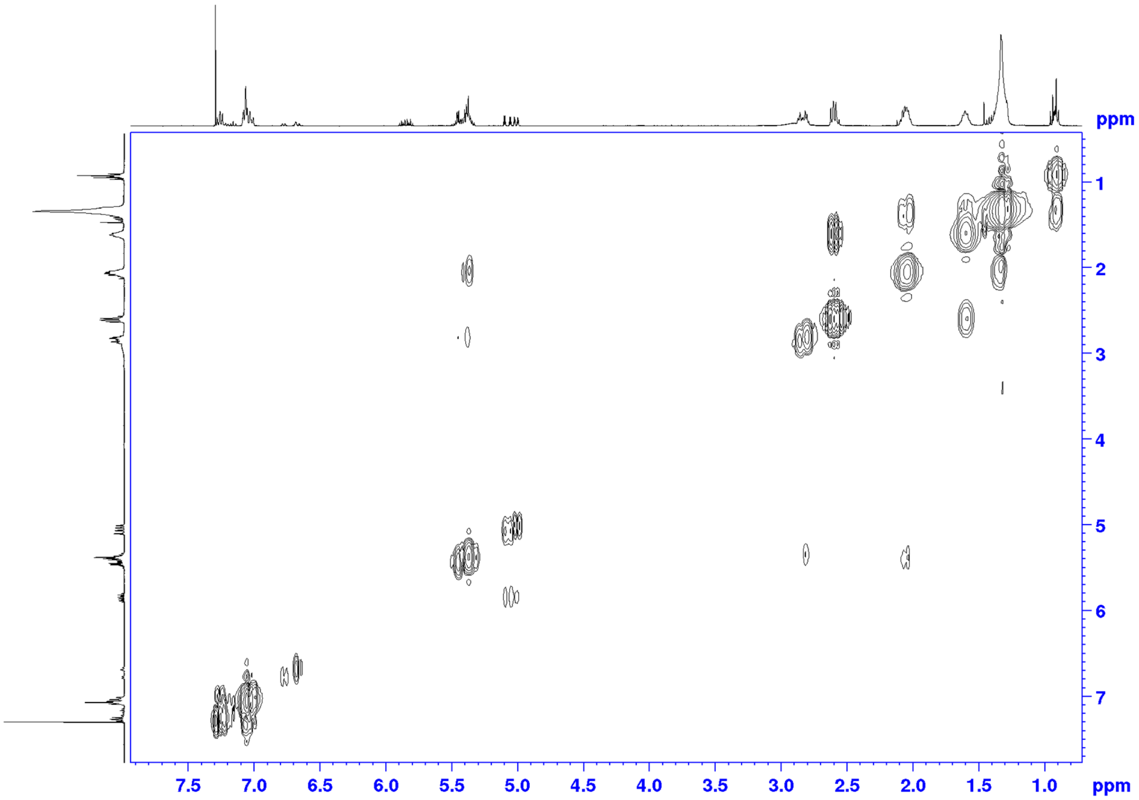

Figure S11: COSY ¹H-¹H of TECP.

II.<u>DSC analyses</u>

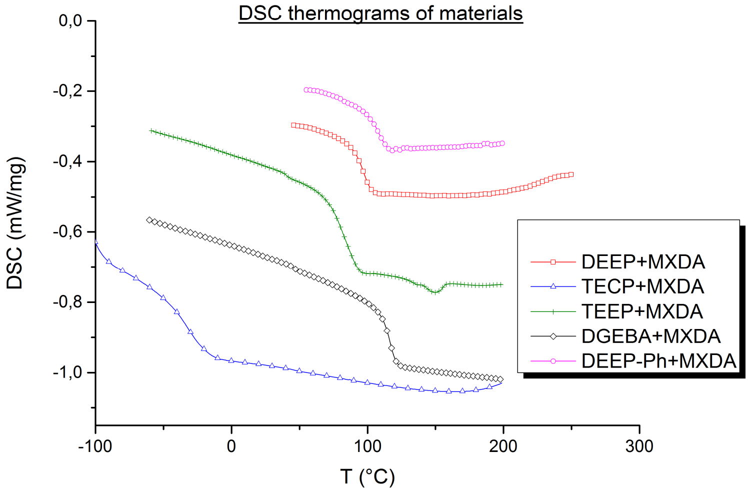

Figure S12: DSC analyses of the materials.
